# Validation of spectral sleep scoring with polysomnography using forehead EEG device

**DOI:** 10.3389/frsle.2024.1349537

**Published:** 2024-05-10

**Authors:** Julie A. Onton, Katherine C. Simon, Allison B. Morehouse, Alessandra E. Shuster, Jing Zhang, Andres A. Peña, Sara C. Mednick

**Affiliations:** ^1^Institute for Neural Computation, University of California, San Diego, La Jolla, CA, United States; ^2^Department of Pediatrics, School of Medicine, University of California, Irvine, Irvine, CA, United States; ^3^Pulmonology Department, Children's Hospital of Orange County, Orange, CA, United States; ^4^Department of Cognitive Sciences, Sleep and Cognition Lab, University of California, Irvine, Irvine, CA, United States

**Keywords:** sleep, spectral scoring, EEG, polysomnography (PSG), mobile EEG device, scoring algorithm, hidden markov model

## Abstract

**Introduction:**

Visual scoring of sleep electroencephalography (EEG) has long been considered the gold standard for sleep staging. However, it has several drawbacks, including high cost, time-intensiveness, vulnerability to human variability, discomfort to patients, lack of visualization to validate the hypnogram, and no acknowledgment of differences between delta and slow oscillation deep sleep. This report highlights a spectral scoring approach that addresses all these shortcomings of visual scoring. Past algorithms have used spectral information to help classify traditional visual stages. The current method used the clearly visible spectral patterns to develop new spectral stages, which are similar to but not the same as visual stages. Importantly, spectral scoring delivers both a hypnogram and a whole-night spectrogram, which can be visually inspected to ensure accurate scoring.

**Methods:**

This study compared traditional visual scoring of 32-channel polysomnography with forehead-only spectral scoring from an EEG patch worn concurrently. The PSG was visually scored by trained technicians and the forehead patch was scored spectrally. Because non-rapid eye movement (NREM) stage divisions in spectral scoring are not based on visual NREM stages, the agreements are not expected to be as high as other automated sleep scoring algorithms. Rather, they are a guide to understanding spectral stages as they relate to the more widely understood visual stages and to emphasize reasons for the differences.

**Results:**

The results showed that visual REM was highly recognized as spectral REM (89%). Visual wake was only scored as spectral Wake 47% of the time, partly because of excessive visual scoring of wake during Light and REM sleep. The majority of spectral Light (predominance of spindle power) was scored as N2 (74%), while less N2 was scored as Light (65%), mostly because of incorrect visual staging of Lo Deep sleep due to high-pass filtering. N3 was scored as both Hi Deep (13 Hz power, 42%) and Lo Deep (0–1 Hz power, 39%), constituting a total of 81% of N3.

**Discussion:**

The results show that spectral scoring better identifies clinically relevant physiology at a substantially lower cost and in a more reproducible fashion than visual scoring, supporting further work exploring its use in clinical and research settings.

## 1 Introduction

Visual sleep scoring has long enjoyed “ground truth” status but visual scoring is not without faults, nor is it the only way to interpret sleep data. Many attempts have been made to use machine learning algorithms instead of visual scoring, but while these algorithms can be as accurate as another visual scorer (Ferri et al., 1989; Stanley, 2023), they have not been accepted as equal to visual scoring. Currently commercially available options advertise that their scoring algorithm can reduce scoring time, but not replace it. This is because in order to validate the automated scoring, the technician must manually scroll through the entire dataset as they would to visually score it anyway. Thus, despite many algorithms and several commercially available options boasting accuracy on par with human scorers, these applications will never be more than supportive technology due to the problem of validating the final hypnogram.

One alternative approach to visual scoring, developed by the first author, is an automated spectral scoring technique which relies on a spectral transformation of the raw EEG into frequencies over time (Onton et al., 2016). This differs from previous algorithms using spectral information because it does not use spectral information to extract what a human would score, but rather reads the spectral information as the stage itself. In other words, spectral scoring allows the spectral data to dictate the sleep stage rather than being used to reverse engineer what a human would see in the raw time domain data. The resulting spectral hypnogram can be quickly visually compared with the corresponding spectrogram image which shows how frequency power and therefore sleep cycles varied across the night. Thus, it has the distinct advantage of providing a visual verification of the final hypnogram results. Visual inspection of the spectrogram can also quickly identify abnormal sleep that may need further attention. The algorithm investigated here uses five frequency bands from a single channel of EEG to separate the night into four possible sleep stages and Wake (Onton et al., 2016). The algorithm was optimized using the easily accessible forehead region, which allows for minimally disrupted sleep in the home environment.

What no other algorithm to date has recognized or dared to consider is that forcing an algorithm to recapitulate visual scoring may blind us from seeing how the brain actually behaves during a night of sleep. In other words, the “ground truth” status that has been given to visual scoring may have been hampering progress toward better understanding of sleep physiology. For example, simply looking at the spectrogram of a night of sleep demonstrates two clearly identifiable deep sleep states, one in the delta range (1–3 Hz) and one in the slow oscillation range (<1 Hz) (Onton et al., 2016). This difference in dominant slow wave frequency is even known to have behavioral differences in the research literature (Mölle et al., 2002; Bersagliere and Achermann, 2010; Kawai et al., 2020), but this distinction continues to be ignored in visual sleep scoring. Spectral scoring, on the other hand, which uses stages based on the dominant frequencies expressed in the data, found that a lack of slow oscillations was significantly more common in a military posttraumatic stress disorder (PTSD) population compared to healthy sleepers (Onton et al., 2018). Visual scoring not only misses some aspects of sleep due to antiquated stage definitions, but it also requires an extremely cumbersome number of electrodes across the scalp that can only be positioned in a laboratory setting. In contrast, spectral scoring requires only one channel from the accessible forehead region to get a rich understanding of the overall structure of the sleep EEG as well as the specific stages encountered during the night. This difference in equipment means not only that a patient can be more comfortable and in their home environment, but also that the cost per recording is drastically reduced. Spectral scoring then allows for the acquired data to be scored objectively using a computer algorithm that is robust yet also based on simple spectral power trends in the data which can be easily validated against the whole night spectrogram. For all these reasons, a shift from visual scoring to spectral scoring could revolutionize sleep medicine and research.

Spectral scoring relies on different information than visual scoring, which results in spectral stages that do not necessarily have a direct visual stage correlate. For example, rather than scoring N1, N2, and N3 stages of non-rapid eye movement (NREM) sleep according to standard visual scoring rules, spectral scoring classifies three dominant patterns of spectral activity during NREM. The three NREM spectral sleep stages are defined as Light (predominance of spindle power), Hi Deep (predominance of 1–3 Hz power), and Lo Deep (predominance of power below 1 Hz). Both Hi and Lo Deep sleep are similar to N3 but are separated because of clear spectral differences between them. In addition, it has been shown in animals that slow oscillations (Lo Deep) have a cortical origin while delta (Hi Deep) has a thalamic origin (Steriade et al., 1993a,b). Electrodermal activity is elevated and oscillating (i.e., “storming”) during Lo Deep sleep, but not during Hi Deep sleep (Onton et al., 2016). Behaviorally, slow oscillations appear to be associated with remembering information while delta may be associated with forgetting (Kim et al., 2019). Slow oscillations can also be enhanced in humans by delivering auditory stimulation at the optimal phase of the slow oscillation, which results in improved memory recall the next day (Ngo et al., 2013). Despite these differences, visual sleep scoring does not differentiate between delta and slow oscillations.

There are several reasons to look beyond the traditional sleep scoring approach for sleep analysis. Spectral scoring provides a more objective, simple, and clear set of rules for scoring that does not rely on human judgment. In visual sleep scoring, there is variability between scorers within a lab, and the even more pronounced problem of variability between labs where the interpretations of the American Academy of Sleep Medicine (AASM) rules may vary. The objective rules in spectral scoring should reduce a degree of this variability. Spectral scoring is fast and requires minimal person-time, a few minutes of computer time compared to several hours of technician time. Additionally, many nuances in the sleep EEG can be detected very quickly by observing the whole-night spectrogram. This could be useful for clinical sleep assessment and could also lead to important discoveries in the research setting. And finally, requiring only one channel of EEG allows for a simple forehead-only device that is more comfortable to wear than current lab or at-home recording devices used for visual scoring.

The current study focuses on the correspondence between traditional visual sleep scoring of full PSG and spectral scoring of EEG from a headband-free, 3-channel, forehead-adherable device recorded concurrently. We expect that this information will encourage use of the spectral sleep scoring technique for rapid, consistent, and more informative sleep reports, as well as a move toward less invasive, at-home recording devices that minimize sleep disruption.

## 2 Materials and methods

### 2.1 Participants

All study activities were approved by the Internal Review Board at the University of California at Irvine and the Army Office of Human Research Oversight. Twenty-six subjects (mean age = 23.2, 15 females) were recruited using word of mouth, social media, or flyers. One subject was dropped due to illness and in six cases the EEG forehead device fell off during the night or was corrupted by artifactual noise indicative of a loose connection. Therefore 19 subjects were used for the final analyses contained in this report. Subjects were 18+ years old, regularly obtaining 7–9 h of sleep per night, habitual bedtime between 10 pm and midnight and habitual wake time between 6 and 8 am and a non-polarized chronotype (Horne-Ostberg Morningness-Eveningness Questionnaire score between 31–69) (Horne and Ostberg, 1976). Subjects were excluded if a sleep disorder was reported or detected, they or a first degree relative was diagnosed with a significant psychopathology, they had a personal history of head injury or loss of consciousness >2 min or seizures, a history of substance abuse, current use of psychotropic medications, or any cardiac, respiratory, or other medical condition that may affect cerebral metabolism. Subjects completed the following questionnaires: (1) Epworth Sleepiness Scale (Johns, 1991), (2) Horne-Ostberg Morningness-Eveningness Quesionnaire (Horne and Ostberg, 1976), (3) Karolinska Sleepiness Scale, (4) Beck Depression Inventory, and (5) General Anxiety Disorder-7 (Spitzer et al., 2006). Subjects were asked to refrain from caffeine for at least 12 h, and alcohol or other stimulants for 24 h before their scheduled sleep time at the lab.

### 2.2 Equipment/setup

The CGX Patch is a small, portable, battery powered EEG recording device (CGX, San Diego, CA) that adheres to the forehead with a single medical-type adhesive with three hydrogel leads at FP1, FP2, and AFz. FP1 serves as the reference, though all three channel differences can be derived (FP1-AFz, FP1-FP2, and FP2-AFz). Data are written to an internal microSD card at 500 Hz with no hardware filters. Each data record is marked with the start and stop internal clock times.

EEG data for the PSG was collected with a 32-channel BrainCap by EASYCAP (Wörthsee, Germany) attached to a Brain Amp Standard (BrainProducts, Munich, Germany) amplifier which collects at 5,000 Hz with a low frequency hardware cutoff of 0.016 Hz. The cap contains 32 channels total, with 3 channels (FP1, FPz, and FP2) that were not used because of the EEG patch placement. All other EEG channels (F3, F4, C3, C4, F7, F8, T7, T8, P7, P8, FT9, FT10, Fz, Cz, COz, PZ, POz, E1, E2, O1, O2, P3, and P4) were collected, along with 2 electrocardiogram and 2 chin electromyogram electrodes. The ground electrode was located between FPz and Fz and the online reference was located between Fz and Cz.

### 2.3 Procedure

**Figure 1:**
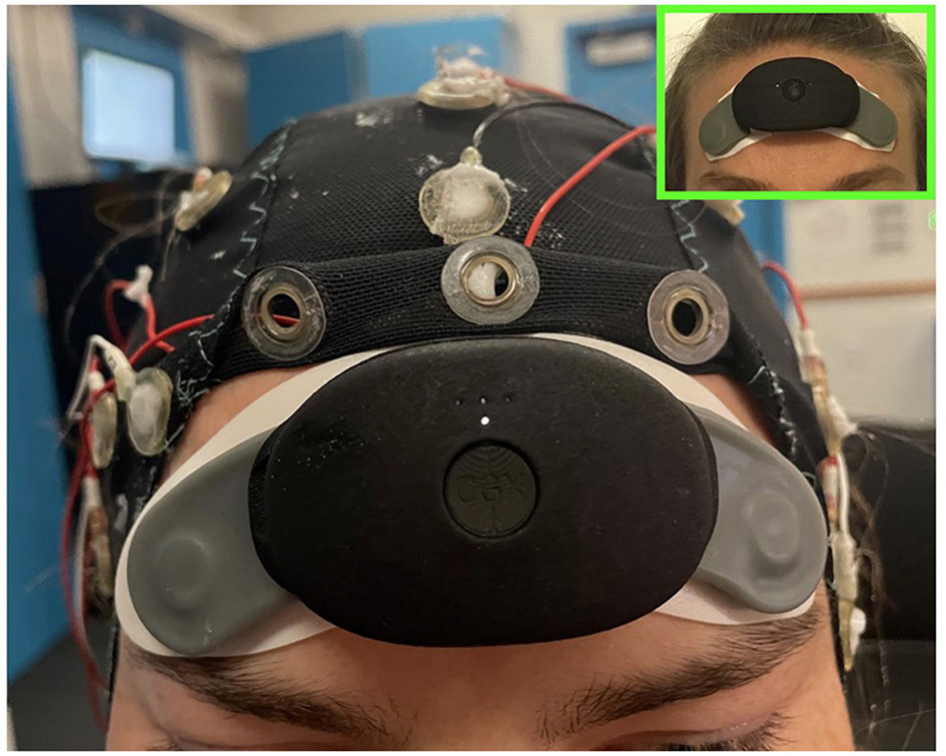
Picture of a subject wearing the CGX patch and EEG cap concurrently in order to collect both forehead EEG for spectral scoring and cap data for visual scoring. In the upper right is a picture of the CGX patch worn alone to show its minimal footprint as a sleep tracking device.

Subjects were fitted with a small, battery-operated forehead EEG recording patch (CGX, San Diego, CA) and then with a full EEG cap that was folded back in the front to accommodate the patch (Figure 1). Subjects slept in a small bedroom, both EEG systems were turned on to record the entire night and the lights were turned off.

### 2.4 Visual scoring

For visual sleep scoring, full PSG cap data were resampled to 256 Hz, filtered between 0.5 and 35 Hz, and re-reference to contralateral mastoid for electrodes F3, F4, C3, C4, E1, E2, O1, and O2 using BrainVision Analyzer 2.0 (BrainProducts, Munich, Germany). Sleep scoring was accomplished by two technicians independently, scoring 30-sec epochs at a time into wake, stage 1 NREM sleep (N1), stage 2 NREM sleep (N2), stage 3 NREM sleep (N3), and REM sleep according to the AASM rules for sleep scoring using HUME, a custom MATLAB (Natick, MA) toolbox. If inter-scorer reliability was <90%, a third reviewer independently scored the sections of discrepancy and the stages with two out of three scores was applied.

### 2.5 Spectral scoring

Details of the data processing have been reported previously (Onton et al., 2016), but the procedure is summarized here. CGX Patch EEG data were imported into Matlab (Mathworks, Natick, MA, USA) where the two original channels referenced to FP1 (FP1-AFz, FP1-FP2) were subtracted to create the third FP2-AFz channel. Each channel was decomposed into frequency amplitude from 0.1 to 100 Hz for each 0.5 sec time point using Morlet wavelet analysis with 3 cycles at the lowest frequency, 30 cycles at the highest frequency and a smooth distribution of cycles at the frequencies in between. This gradient of cycles produces an optimal trade-off between time and frequency resolution for each frequency. The real portion of the amplitudes was computed by multiplying the complex number by its conjugate and then converted to decibels (dB) by the formula 10^*^log10 (amplitude). The baseline was calculated by averaging across the entire night except noise epochs defined by extreme root mean squared or total power values. The baseline spectrum was subtracted from the spectrum at each time point to create the spectrogram used for spectral band calculations. The dominant frequency display was derived from the baselined spectrogram by marking a dot at the frequency with the maximum power value at each time point. The display spectrogram was smoothed using a moving 40-sec window for better visualization of the macroscopic patterns. Line noise frequencies between 50 and 70 Hz were masked by assigning the power at surrounding frequencies for the visual spectrogram and they were ignored for calculation of the Wake power band.

**Figure 2:**
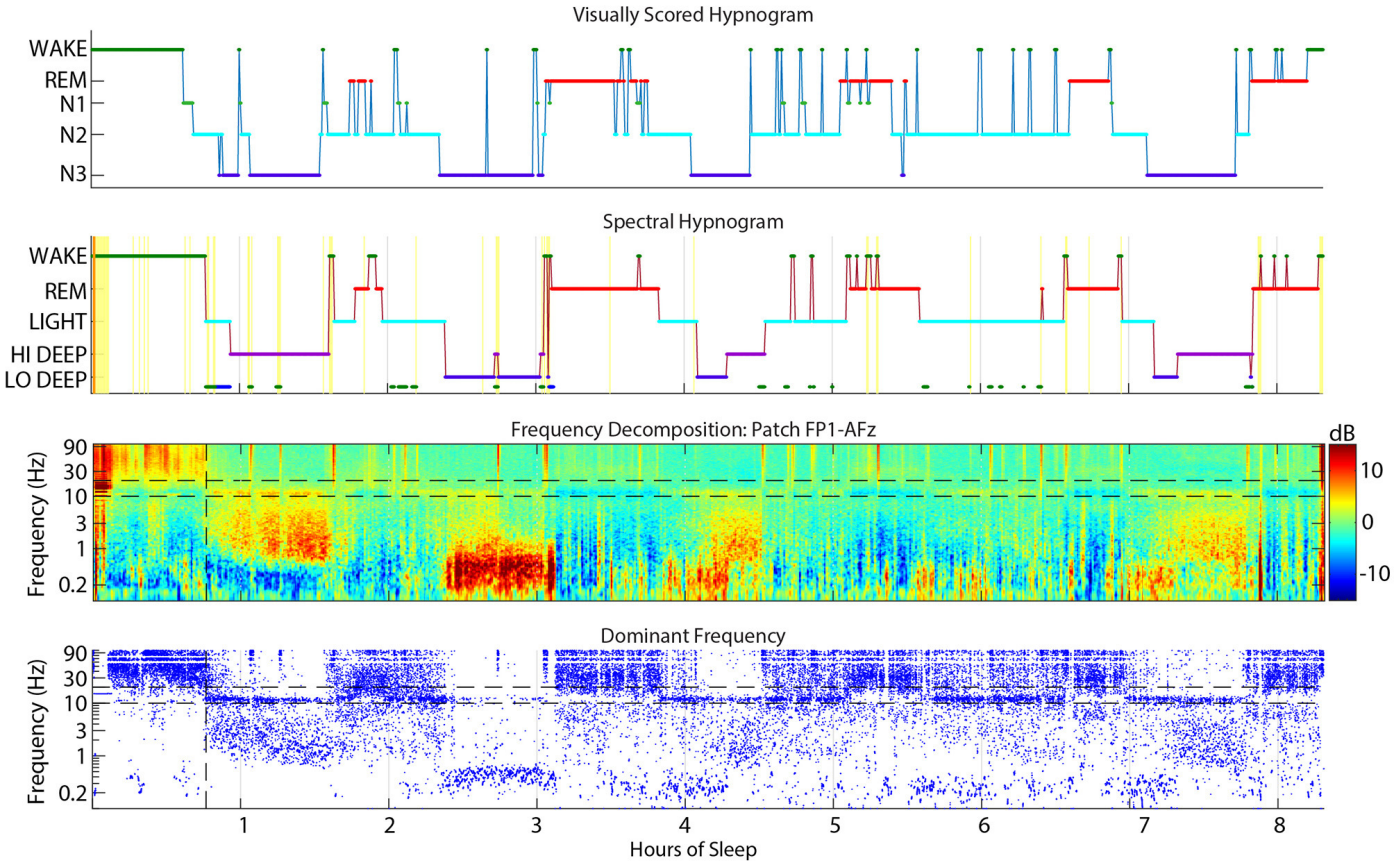
Example sleep report showing general alignment of cap visual scoring **(top)** and CGX patch spectral scoring **(second)**. The **third** panel is the whole-night spectrogram showing relative dB power (baseline across the whole night removed). The **bottom** panel shows the dominant frequency from the spectrogram at each 0.5 sec window, which helps to highlight low amplitude spectral power in higher frequencies like low beta during REM sleep.

Each patch channel (FP1-AFz, FP2-AFz and FP1-FP2 [“FF”]) was submitted separately to the sleep scoring algorithm which converts the data from the whole night into a full spectrogram from 0.1–100 Hz then averages five spectral bands for scoring. The details of the spectral sleep scoring algorithm have been published previously (Onton et al., 2016). But to summarize, each 30-sec time window is represented with 5 frequency bands which define 5 stages of sleep: Wake (40–95 Hz), REM (~17–26 Hz), Light (~11–15.5 Hz), Hi Deep (1–3 Hz), and Lo Deep (0.1–1 Hz). While REM sleep is also associated with frontal theta power (Nishida et al., 2009), it is not consistent enough to use for accurate spectral scoring. This information was modeled as a HMM and refined with estimation maximization (Rabiner and Juang, 1986). For each 30-sec epoch, the conditional probability of the subject being in each stage was calculated and the stage with maximal probability was assigned. The following modifications have been added since the original publication of the algorithm that account for several common ambiguities that can lead to mis-scoring. Both REM and Light peak frequencies are refined after a first pass scoring and the ranges set to −1 to +1.5 Hz around the peak spindle frequency for Light, and −2 to +2 Hz around the peak frequency for REM. The function then conducts a series of post-HMM corrections for irregular sleep expressions. For example, some sleep records show high power in the Wake frequency band (40–95 Hz) while the spindles and low frequency power clearly shows NREM sleep. Thus, if the algorithm scored these segments as Wake, the post-correction tests will change them back to NREM based on the lower frequency sleep rhythms. Other automatic corrections were: (1) changing Lo or Hi Deep to REM or Wake based on a high beta-to-spindle ratio along with relatively low total spindle power, (2) Lo or Hi Deep changed to Wake if total power is high and spindles are relatively low (indicates very noisy data that is not sleep), (3) Lo or Hi Deep changed to Light when spindle power is greater than low frequency power, and (4) Wake to Hi Deep or Light if delta or spindles are sufficiently high, indicating NREM sleep, and it is after initial sleep onset. On very clean sleep, these tests are unnecessary, but on more complicated records they are essential to accurate scoring. The colored dots at the bottom of the hypnogram denote the changed epochs (e.g., Figure 2). Each colored dot indicates the sleep stage from which the epoch was changed (green = Wake, red = REM, cyan = Light, blue = Hi or Lo Deep). The current report uses a 30-sec scoring time window for consistency with PSG scoring, but in practice this window can be decreased to about 10-sec windows with little change in the spectral hypnogram. For any duration below 10 seconds the hypnogram begins to jump frequently between stages when there are momentary spindle or delta bursts, for example, that alternately achieve dominance in very short time windows.

### 2.6 Visual and spectral scoring alignment

The CGX Patch and cap data records were aligned as accurately as possible based on the computer clock start time of the EEG cap file and the start time of the forehead patch. The overall alignment of the files can be assessed in Figure 2 which shows an example night with visual scoring (top panel), spectral scoring (second panel), spectrogram (third panel), and dominant frequency (bottom panel).

### 2.7 Comparison of visual and spectral scoring

Visual and spectral hypnograms were compared by finding the number of epochs scored in each stage matching (e.g., N2 and Light, or N2 and Hi Deep, etc.). This information populated a confusion matrix between the 5 stages of visual scoring (wake, REM, N1, N2, and N3) and the 5 stages of spectral scoring (Wake, REM, Light, Hi Deep, and Lo Deep). In general, a confusion matrix is a representation of the performance of a classification model, displaying the true and predicted values for each class in a table for easy assessment of model accuracy. In this case, there is no actual true or predicted values, we are simply offering a concise way to view the agreements and disagreements between the methods. In order to quantify the scoring confusions across subjects, confusion matrices for each subject were converted to percentages in each direction (rows and columns) and pooled across subjects to obtain the medians for each visual/spectral stage pair.

## 3 Results

### 3.1 CGX patch derivation differences and channel selection

All CGX Patch channels returned similar percentages in each voxel of the confusion matrix (average of ±1.5 standard deviations between all CGX Patch channels for both visual to spectral and spectral to visual; range: 0.1–7.0). Some voxels had larger differences between channels, such as visual wake to spectral Wake (45%, 48%, and 57%, for FP1, FP2, and FF, respectively). Low agreement between channels in the percentage of Hi Deep scored as N3 in (56%, 61%, and 70% on FP1, FP2, and FF, respectively) were likely due to some incorrectly scored delta activity on FP1 during wake before sleep onset and prolonged wake before lights on with FP1 and FP2 spectral scoring. It is unclear if this activity is eye movements or true brain-derived activity, but these results suggest that much of it appears to be canceled out on the FF derivation. The other area of disagreement was in the designation of N3 as Hi or Lo Deep sleep. The FF derivation found 54% of N3 epochs to be Hi Deep and only 27% as Lo Deep. In contrast, FP1 and FP2 found 42–43% of N3 epochs as Hi Deep while finding 38–39% of epochs to be Lo Deep. This discrepancy is likely attributable to the attenuation of slow oscillations on the FF derivation due to the generally central origin of slow oscillations. Because of the importance of Lo Deep to the spectral scoring mechanism, this report will focus on the scoring of FP1 and FP2 which were on average only ±65 epochs different from each other (range: 1–338). To simplify the presented results and to not favor one side over the other, the number of epochs from the confusion matrices for FP1 and FP2 were averaged.

**Figure 3:**
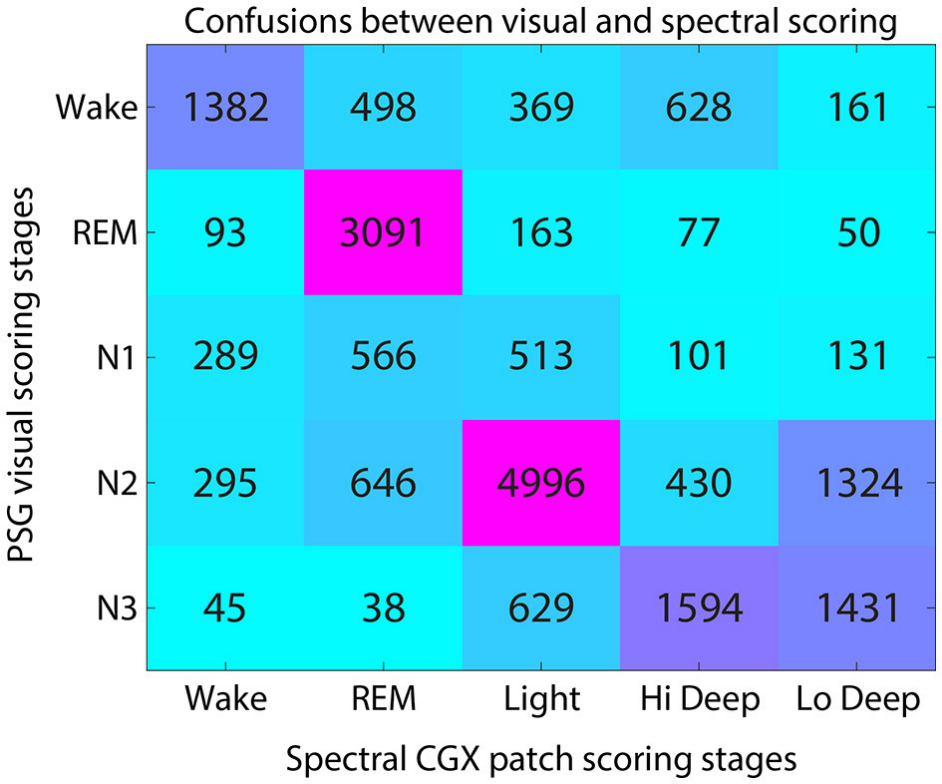
Confusion matrix for visual and spectral scoring techniques. Numerals indicate the number of total epochs across subjects that were scored as each visual-spectral scoring combination.

Figure 3 shows the average confusion matrix for FP1-AFz and FP2-AFz derivations across subjects. Each voxel shows the number of epochs scored as the given visual stage (row) and the given spectral stage (column).

The highest agreement between scoring systems was in the visual-to-spectral REM assignment. For epochs that were visually scored as REM, the spectral algorithm also identified 89% of these epochs as REM (Figure 3). In the converse direction (along the column), spectral REM was scored as REM by visual scorers 64% of the time. The confusions in this direction were equally divided among N1, N2 and wake, accounting for 12% of epochs each (Figure 3).

Hi and Lo Deep sleep accounted for 81% of N3 epochs, while Light sleep accounted for 17% of N3 epochs (Figure 3). Visual N3 epochs were scored mostly as Hi Deep (42%) but were also scored frequently as Lo Deep sleep (39%). In the opposite direction, Hi Deep was scored as N3 59% of the time, and N2 16% of the time. In contrast, epochs scored as spectral Lo Deep were scored as N3 only 47% of the time and N2 43% of the time.

Visually scored N2 was most often scored as spectral Light (65%), with Lo Deep being the next most common spectral score at 17%. In the opposite direction, spectral Light was scored as N2 74% of the time, meaning that Light rarely aligns with any other visually scored stage, with the next highest confusion being for N3 9% of the time (Figure 3).

Epochs scored as N1 were approximately equally scored as REM (34%) or Light (32%), with somewhat fewer epoch scored as Wake (19%). Since there is no spectral equivalent of N1, the observed confusions are consistent with being a transitional state between spectral Wake, REM, and Light sleep.

Finally, visual wake was scored as spectral Wake 47% of the time, with the main confusions being REM (18%) and Light (13%). In the opposite direction, spectral Wake was scored as visual wake 65% of the time, with N2 (14%) and N1 (14%) being the most common confusions. Interestingly, spectral Wake was visually scored as REM only 4% of the time, suggesting that the spectral algorithm rarely mistakes REM for Wake.

### 3.2 Stage confusion ranges

**Figure 4:**
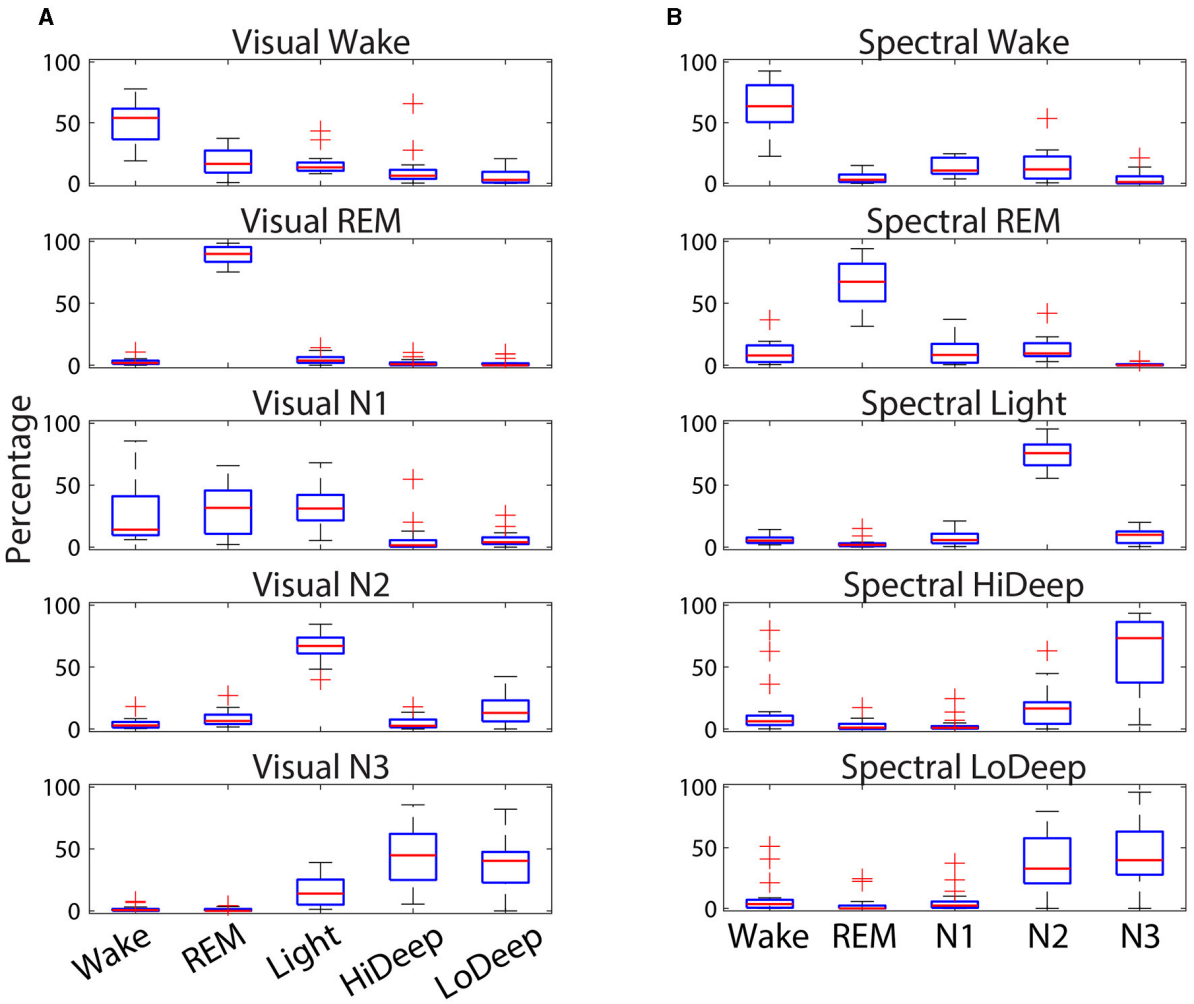
Confusion percentages (y-axis) across subjects of CGX patch spectral stage designations (x-axis) for each cap-scored visual stage **(A)**. Percent confusions (y-axis) across subjects of cap-scored visual stage designations (x-axis) for each patch-scored scored spectral stage **(B)**. Red lines indicate the median, while the boxes represent the 25^th^ and 75^th^ percentiles and plus symbols show outliers.

Figure 4 shows the numerical ranges of stage confusion percentages across subjects. Visual REM was not only the stage with the most consistent agreement with spectral REM, but it also showed very little deviation across subjects within any of the spectral stages. Spectral Light was also consistently scored as visual N2 with little deviation across subjects. The most deviation occurred in visual N1, which has no direct correlate in spectral scoring. Spectral Hi Deep had several outliers in which Hi Deep was scored during visual wake. This can happen when a subject is awake for a long time either before sleep onset or at the end of the night. During these times subjects may express high delta power (that would normally be Hi Deep) along with high gamma power (indicating Wake) that is sometimes scored as Hi Deep. Hi Deep also had high variance for visual N3 scoring which is probably due to the inherent difference in scoring rules between N3/N2 and Hi Deep/Light such that Hi Deep might be scored as N2 more frequently in some subjects if delta power is particularly low.

### 3.3 Stage duration ranges

**Figure 5:**
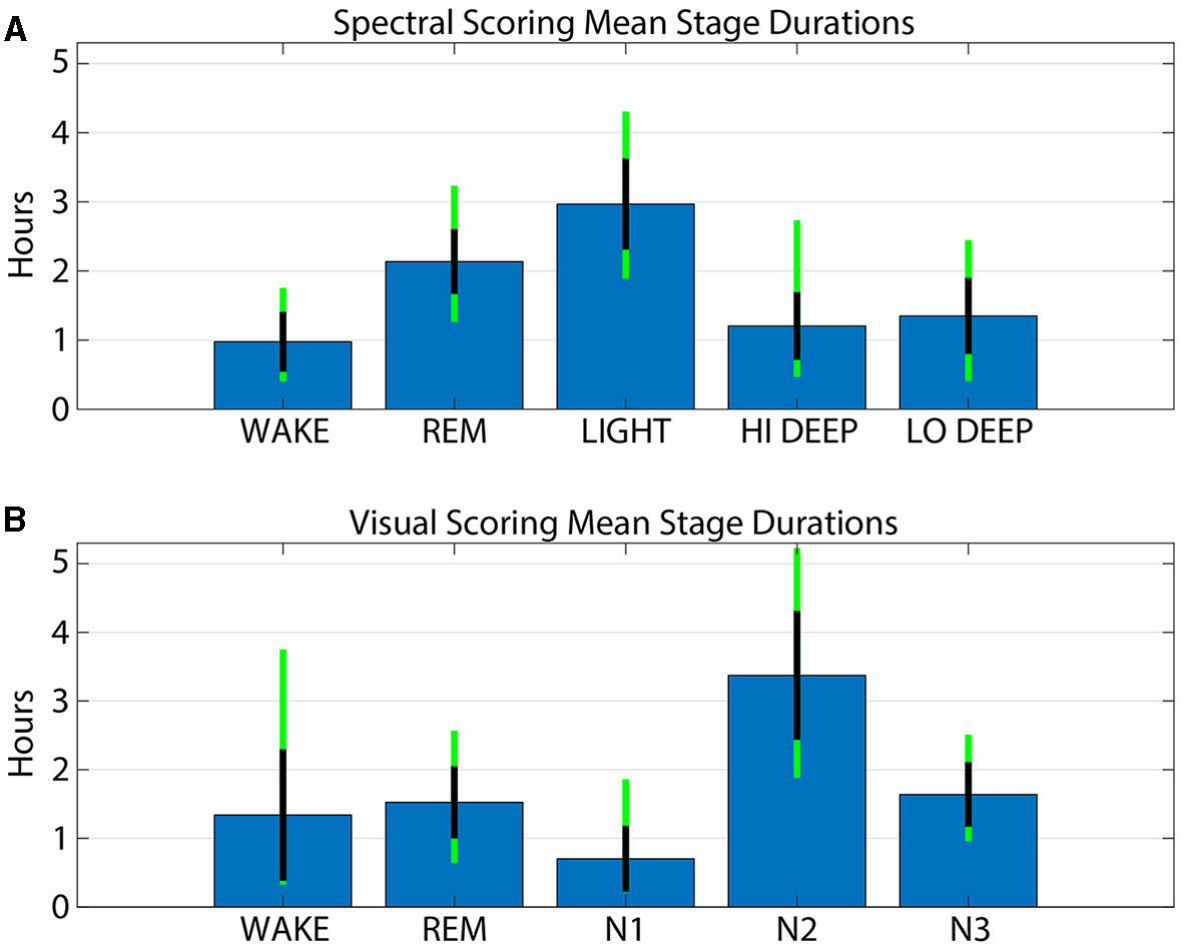
Average stage durations for patch spectral scoring **(A)** and cap visual scoring **(B)**. Blue bars show the mean number of hours for each stage and method. Black lines indicate the standard deviation across subjects and the green line extensions indicate the maximum and minimum values across subjects.

Figure 5 shows the average time spent in each stage for both spectral and visual scoring. The spectral scoring technique scored an average of 30 min more REM than visual scoring. Visual scoring scored about 20 min more wake than spectral scoring. Light and N2 were the most commonly scored stages, with an average of 3.0 h in spectral scoring and 3.4 in visual scoring, respectively. Combined Hi and Lo Deep sleep produced an average of 2.6 h per night, which is far greater than the average 1.6 h of N3 from visual scoring. This owes to the fact that visual scorers sometimes scored N2 for both Hi (17%) and Lo (37%) Deep sleep.

## 4 Discussion

This report is the first to directly compare spectral scoring from a forehead patch device with visual scoring from full PSG recorded simultaneously. The results demonstrate that spectral scoring aligns with high agreement for REM (89%), with only 4% of spectral REM scored visually as wake. This finding suggests that spectral scoring rarely mistakes REM for wake, which is a common error for many sleep scoring algorithms (Cellini et al., 2015). N3 is mostly (81%) described spectrally as either Hi or Lo Deep sleep, as expected, though Light claimed some of these epochs when delta activity was lower power than spindle activity. Spectral Light was scored as N2 74% of the time, meaning that Light is highly similar to the N2 state. These findings underscore the similarities and intuitive associations of spectral and visual scoring. These and the more nuanced findings below now allow for a movement away from visual scoring and toward spectral scoring whenever possible.

**Figure 6:**
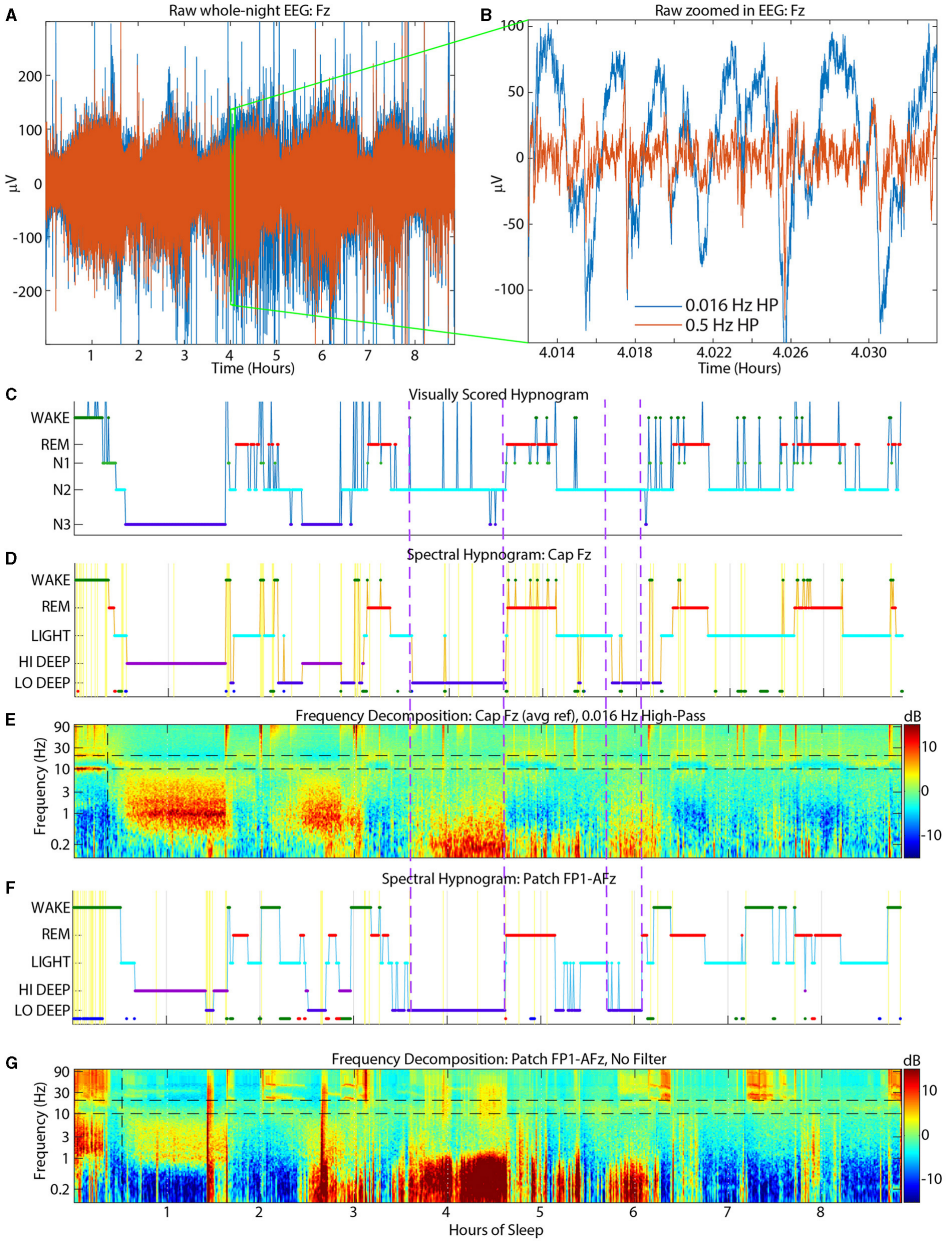
Example sleep report showing strong Lo Deep sleep at both cap Fz **(D, E)** and patch FP1-AFz **(F, G)** around 4 h that is nearly completely scored as N2 by the visual scorers **(C)**. Top panels show raw cap Fz EEG data [**(A)** = whole night, **(B)** = zoom into **(A)**]. Blue traces have a hardware high-pass of 0.016 Hz and red traces have an additional software high-pass of 0.5 Hz for visual scoring. Dashed purple vertical lines indicate time periods when the slow oscillations were scored as N2 by cap visual scorers. HP, High-pass.

N2 was scored as Light only 65% of the time, meaning N2 epochs include other spectral scores besides Light (whereas Light was mostly scored as N2). Oddly, the most common confusion for N2 was Lo Deep, which is likely due to the high-pass filter above 0.5 Hz applied prior to visual scoring. This filter may attenuate much of the activity under 1 Hz which contains the large amplitude slow oscillations that constitute Lo Deep sleep. If delta activity were not simultaneously elevated during a Lo Deep epoch, then a visual scorer might not detect slow waves at all and therefore score N2. An example of this phenomenon is shown in Figure 6 which shows strong slow oscillation activity around 4 h on both patch channel FP1-AFz (F/G) and cap channel Fz before 0.5 Hz high-pass (D/E) that was ultimately scored as visual N2. This figure also shows the raw EEG data from the cap at Fz (A) after the hardware high-pass filter of 0.016 Hz (blue traces) and after the additional 0.5 Hz filter used for scoring (red traces). In this panel it is clear how much amplitude is lost with the addition of the 0.5 Hz filter. The zoomed in panel (B) shows in detail how slow oscillations can be abolished after 0.5 Hz high-pass filtering, leading the visual scorer to assign N2 if delta power is not also strong during that epoch. For a more comprehensive idea of the power differences in the slow wav e sleep range, sleep reports from all subjects with spectrograms from CGX channel FP1-AFz and cap channel F3 are included in the Supplementary material. Another possible reason for the discrepancy may be that slow oscillations are essentially K-complexes (Amzica and Steriade, 1997) which, in the absence of clear delta waves, woul d be indicators of N2 (Silber et al., 2007). Thus, the combination of high-pass filtering, and minimal delta power could make remaining slow oscillations and ongoing spindle activity appear to the visual scorer as N2-related activity.

### 4.1 Visual wake scored as light

**Figure 7:**
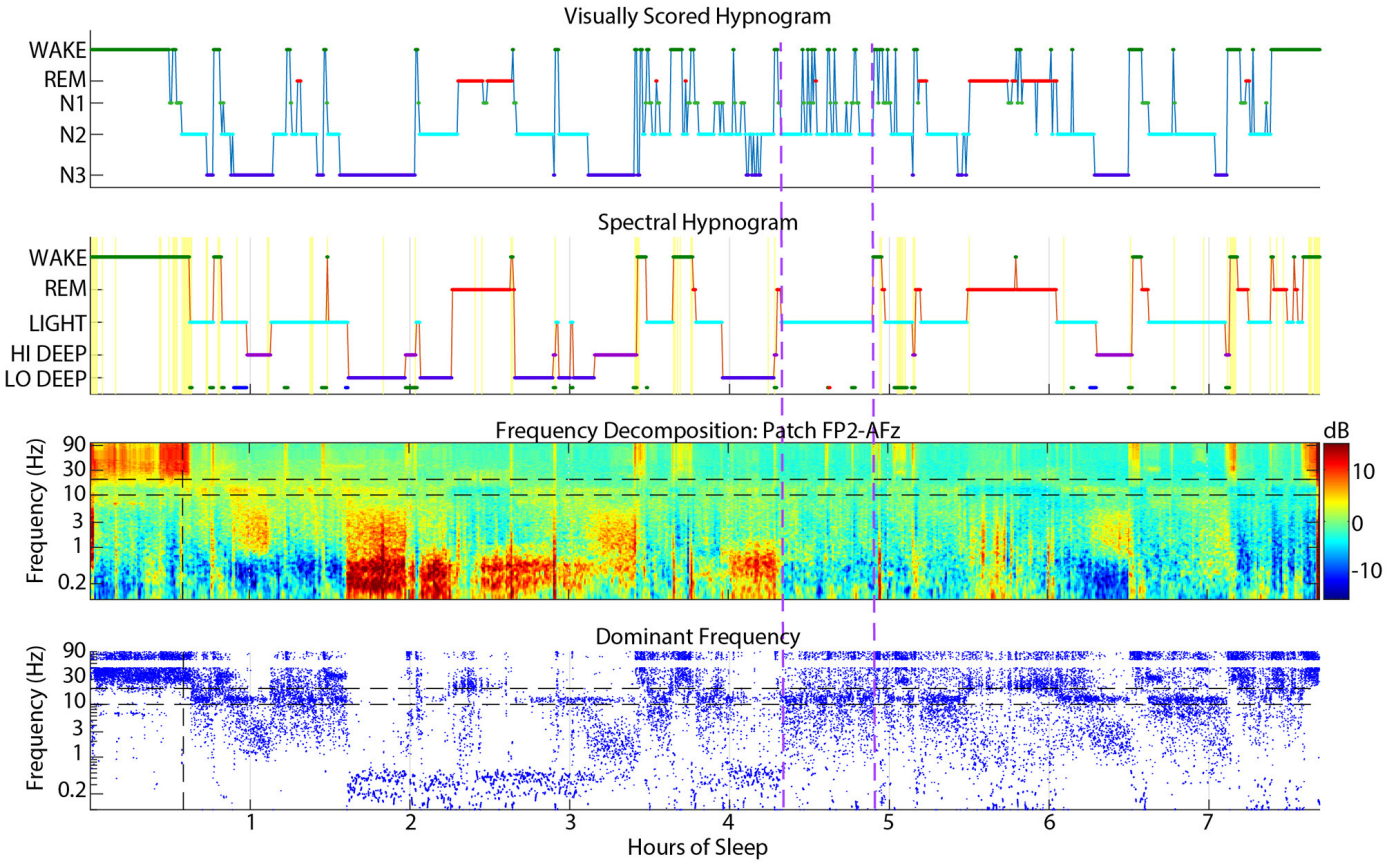
Example sleep report showing a subject whose spectral Light sleep was scored as visual N2 with frequent interruptions into visual N1 or wake (between vertical dashed purple lines) that are rarely justified in the spectrogram or dominant frequency.

Visual wake was scored as spectral Light about 13% of the time, which may be due to visual scoring rules requiring clear sleep spindles lasting at least 0.5 seconds to be scored as N2 (Silber et al., 2007). Alternatively, subjects with a low and/or wide sleep spindle frequency range may appear to have activity in the alpha range which would signal wake or N1. An example of a subject with frequent interruptions of N2 into wake or N1 is shown in Figure 7. These excursions into wake/N1 were not detected by spectral scoring and there is little evidence in the spectrogram or dominant frequency display to justify more than a couple momentary arousals at most.

### 4.2 Spectral REM scored as N2

**Figure 8:**
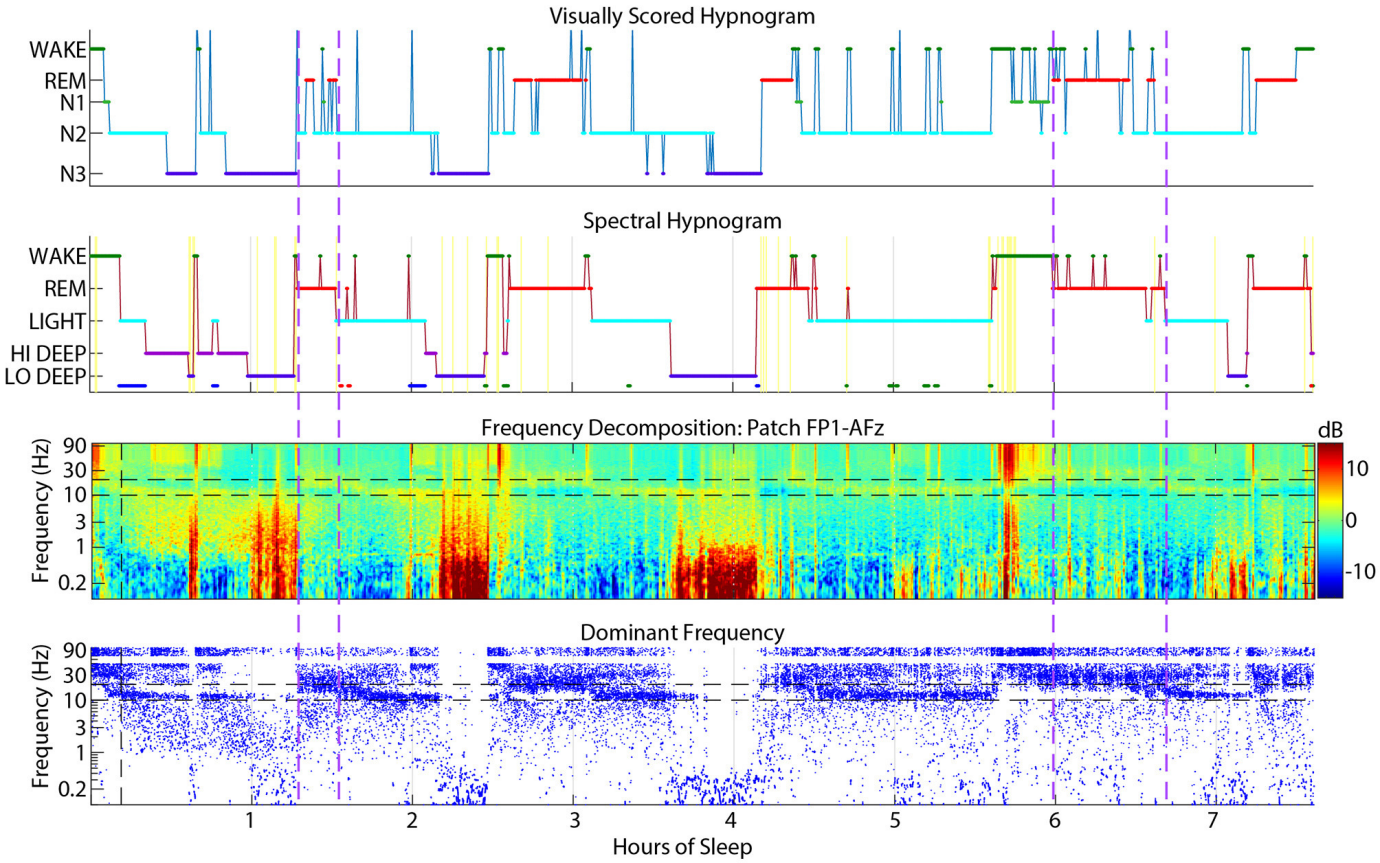
Example sleep report showing a subject whose patch spectral REM sleep was sometimes scored as N2 by visual cap scoring (between vertical dashed purple lines). Spectral REM stages are also often longer than the corresponding visual REM stage. Cap visual scoring frequently assigns N2 instead of REM at the transitions to and from REM.

Spectral REM was scored as visual N2 about 12% of the time, which was one reason for the lower correspondence in REM in this spectral to visual direction. These confusions usually occurred near the boundaries of REM periods, meaning that the spectral method scored REM slightly earlier and often extended longer than visually scored REM. Brief excursions to N2 from REM also occurred during the middle of visual REM periods. Figure 8 shows an example subject with many instances of N2 during REM periods that were mostly scored spectrally as continuous REM. The first REM cycle around 1.5 h might have excessive excursions into N2 because of a dip in the REM beta frequency into the spindle range, which might have looked like N2 to the visual scorer. Though when observing the spindle power band throughout the night, it is clear that this REM beta activity did not enter the actual spindle range for this subject. The REM period after 6 h also contained many N2 epochs that spectral scoring found to be continuous REM sleep, presumably because of the clear absence of spindles in the spectrogram. During this period, the dominant frequency panel (bottom panel, blue dots) demonstrates that low beta power was not disrupted except a brief excursion into spindle power that the spectral algorithm also scored as Light.

### 4.3 Visual and spectral wake confusions

**Figure 9:**
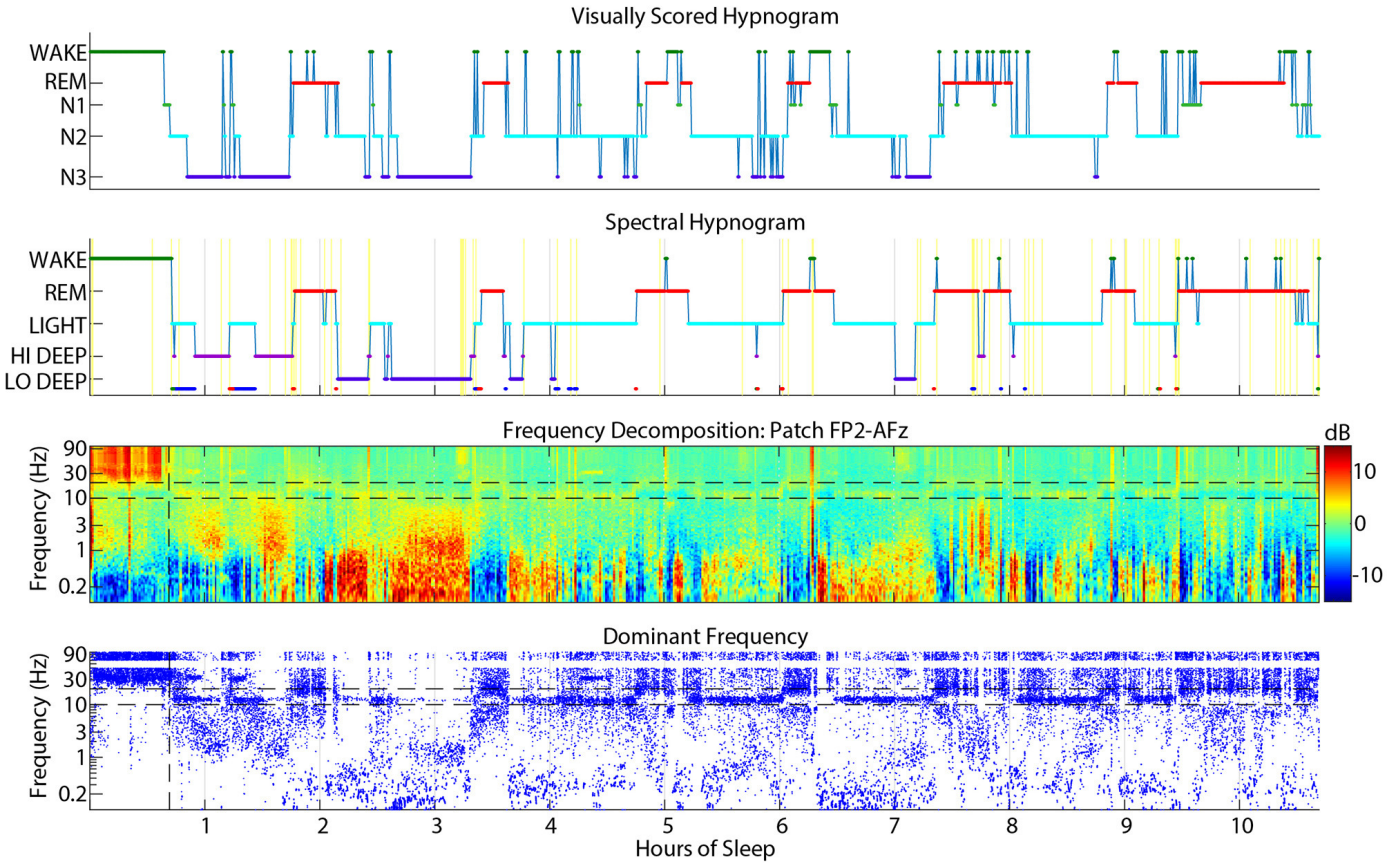
Example sleep report showing frequent visual cap-scored wake epochs while spectral patch scoring was more consistently REM or Light.

Wake scoring agreement in either direction was fairly low, but visual wake scored as spectral Wake was especially low at only 47%. The main confusions were for REM (18%) and Light (13%). This phenomenon may be due to frequent jumps from visual REM or N2 to wake that were scored more continuously as REM or Light by the spectral scoring algorithm (Figure 9). Due to visual scoring rules, it is sometimes necessary to score individual epochs within a larger REM or N2 stage as wake if there is evidence of posterior alpha. In contrast, the spectral HMM algorithm tends to favor continuity because of the probabilistic nature of the HMM function. In the example shown in Figure 9, frequent jumps to wake do not seem justified given the relatively low amplitude gamma activity in the spectrogram during these jumps to visual wake.

### 4.4 Spectral scoring as an alternative to visual scoring

This report is by no means the first automated sleep scoring algorithm, nor the first to use spectral information for scoring purposes (Koley and Dey, 2012). It also does not show the highest overall accuracy, as previous reports have shown 84% (Zhao et al., 2022) and 89% (Ghimatgar et al., 2019) overall accuracy across all sleep stages. However, there are several reasons that spectral scoring may be superior to these prior algorithms. First, they were designed to mimic visual scoring, which as this report has highlighted may itself be flawed, not to mention inconsistent. They were not letting the data tell them what features appear in consistent patterns across the night, but how they can manipulate the data to give the answer that a human would. In contrast, spectral scoring is simply translating the visible patterns that appear after frequency transformation into endogenous stages of sleep. Second, the published methods are extremely complicated and have no visual outputs to let the clinician know how each stage was derived. On the other hand, spectral scoring outputs a spectrogram that shows the clinician the EEG patterns from which the hypnogram was derived so that they may quickly verify the accuracy of the hypnogram. Finally, spectral scoring differentiates between the delta and slow oscillation frequency ranges which may lead to important discoveries and novel diagnoses such as the finding that patients with PTSD were lacking Lo Deep sleep (Onton et al., 2018). This phenomenon is unlikely to be exclusive to this population as Lo Deep quantity was not found to correlate with symptoms of PTSD. Thus, sleep medicine may be able to advance by incorporating Hi and Lo Deep sleep into diagnoses and treatment outcomes.

Even though spectral stages are easy to interpret and clearly defined, this report provides detailed comparisons between spectral and visual scoring for sleep clinicians or researchers to translate their prior knowledge of visual scoring to spectral scoring interpretation. A move to exclusive spectral scoring may occur slowly over time, but in the short term there are many applications that can easily use spectral scoring immediately. For example, spectral scoring could be used to compare sleep quality from before to after a treatment since spectral scoring would be compared to itself and the translation from visual scoring is less critical. In this case, spectral scoring from a single forehead channel would allow for collection of several nights from each treatment phase while still being able to score the data with less time and expense. In any research or clinical situation, the ability to collect several nights from each subject would greatly enhance our understanding of sleep. Up to now, the cost-prohibitive and uncomfortable nature of full PSG analysis has prevented analysis of naturalistic sleep within subject over more than two nights. Sleep clinics could also benefit from this technique by providing more information to the clinician at intake that could steer optimal treatment, and post-treatment follow-up could provide objective evidence of treatment efficacy.

While any EEG device collecting from the forehead would suffice for spectral scoring, the CGX patch device used in this study is uniquely designed to adhere directly to the forehead without the need for a strap. This is a notable advancement because headbands, even when worn relatively loosely, are a slight distraction to normal sleep. On the other hand, the CGX patch device has a slim casing in the middle of the forehead and flexible side flaps that do not give the feeling of sagging, nor does it cause obstruction to side sleeping. It also contains three leads within the device that allow for three derivations. Importantly, it is still possible to detect high amplitude slow oscillations because of the central and lateral placements of electrodes. And the central to lateral derivations allow for investigation of lateralized activity that may be of clinical importance. The CGX patch appears to be the most powerful and comfortable option for low-profile sleep EEG collection to date.

While visual scoring and full PSG will still retain its place in sleep labs when full-head and auxiliary measures are necessary to diagnose patients with complicated sleep disorders, spectral scoring is a powerful tool that could revolutionize sleep research and medicine.

## 5 Limitations

This analysis is limited in that the patch and cap EEG recordings were not exactly synchronized, but rather aligned to the nearest minute or so. Therefore, it is to be expected that the 30 sec epochs used for sleep scoring and confusion matrices may not be referring to the exact same moments in time. This may create artificial divergences between visual and spectral scoring during sleep transitions or during particularly chaotic periods. However, due to the relatively continuous nature of sleep stages, we believe that the comparisons made in this report are substantially correct.

**Figure 10:**
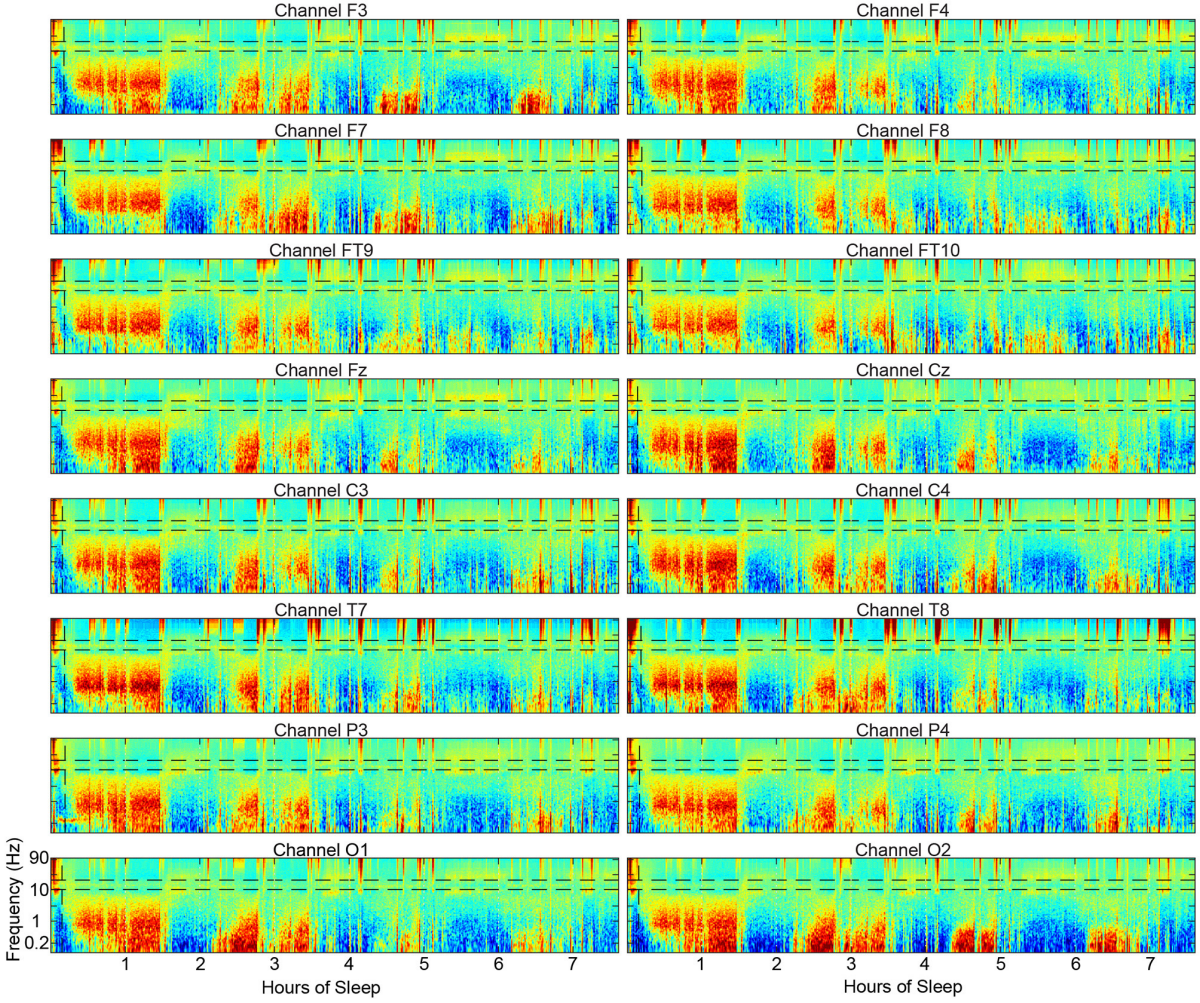
Example spectrograms from 16 scalp electrode locations across the scalp from a single subject showing the substantial similarities in the overall structure of the spectrograms.

One limitation of the spectral scoring method is that it only scores one channel at a time and therefore does not incorporate data from the whole head as visual scoring does. However, spectral scoring can be applied to all channels from a full cap separately and compared as spectrograms and hypnograms to build a whole-head picture. This approach may uncover interesting aspects of the sleeping brain that have not yet been appreciated without a visual representation (Figure 10).

Spectral scoring does not use the alpha band to detect Wake, which is particularly useful in visual scoring where posterior electrodes are present. The reason for not using alpha in spectral scoring is that in frontal electrodes, for which the algorithm was optimized, alpha is not always detectable during Wake while gamma is prominent all over the scalp during Wake. If a posterior channel were used for spectral scoring, it is conceivable to use the presence of alpha as a defining feature of Wake EEG in addition to gamma.

## Data availability statement

The raw data supporting the conclusions of this article will be made available by the authors, without undue reservation.

## Ethics statement

The studies involving humans were approved by Internal Review Board at the University of California at Irvine. The studies were conducted in accordance with the local legislation and institutional requirements. The participants provided their written informed consent to participate in this study.

## Author contributions

JO: Conceptualization, Data curation, Formal analysis, Funding acquisition, Investigation, Methodology, Project administration, Resources, Software, Validation, Visualization, Writing – original draft, Writing – review & editing. KS: Data curation, Investigation, Project administration, Writing – review & editing. AM: Data curation, Investigation, Project administration, Writing – review & editing. AS: Data curation, Investigation, Project administration, Writing – review & editing. JZ: Data curation, Investigation, Project administration, Writing – review & editing. AP: Data curation, Investigation, Writing – review & editing, Project administration. SM: Data curation, Funding acquisition, Investigation, Supervision, Writing – review & editing.
