# Supplementary figures and images for: Validation of spectral sleep scoring with polysomnography using forehead EEG device
Source: Front Sleep. 2024 May 10;3:1349537. doi: 10.3389/frsle.2024.1349537 (PMC12713971; doi:10.3389/frsle.2024.1349537)

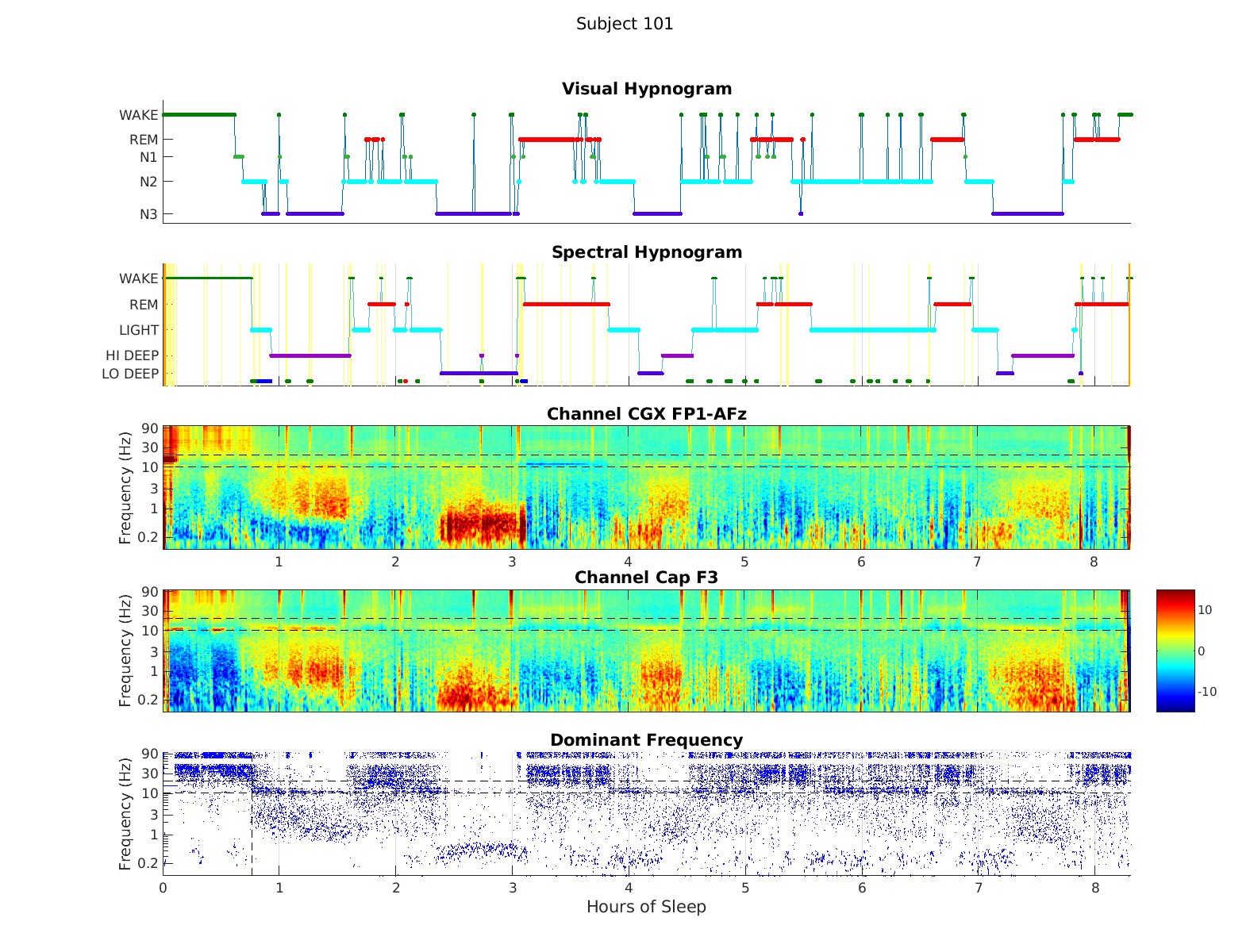

Supplement: Supplementary file 1 [file Data_Sheet_1.ZIP › FP1/101_FP1-F3.jpg]

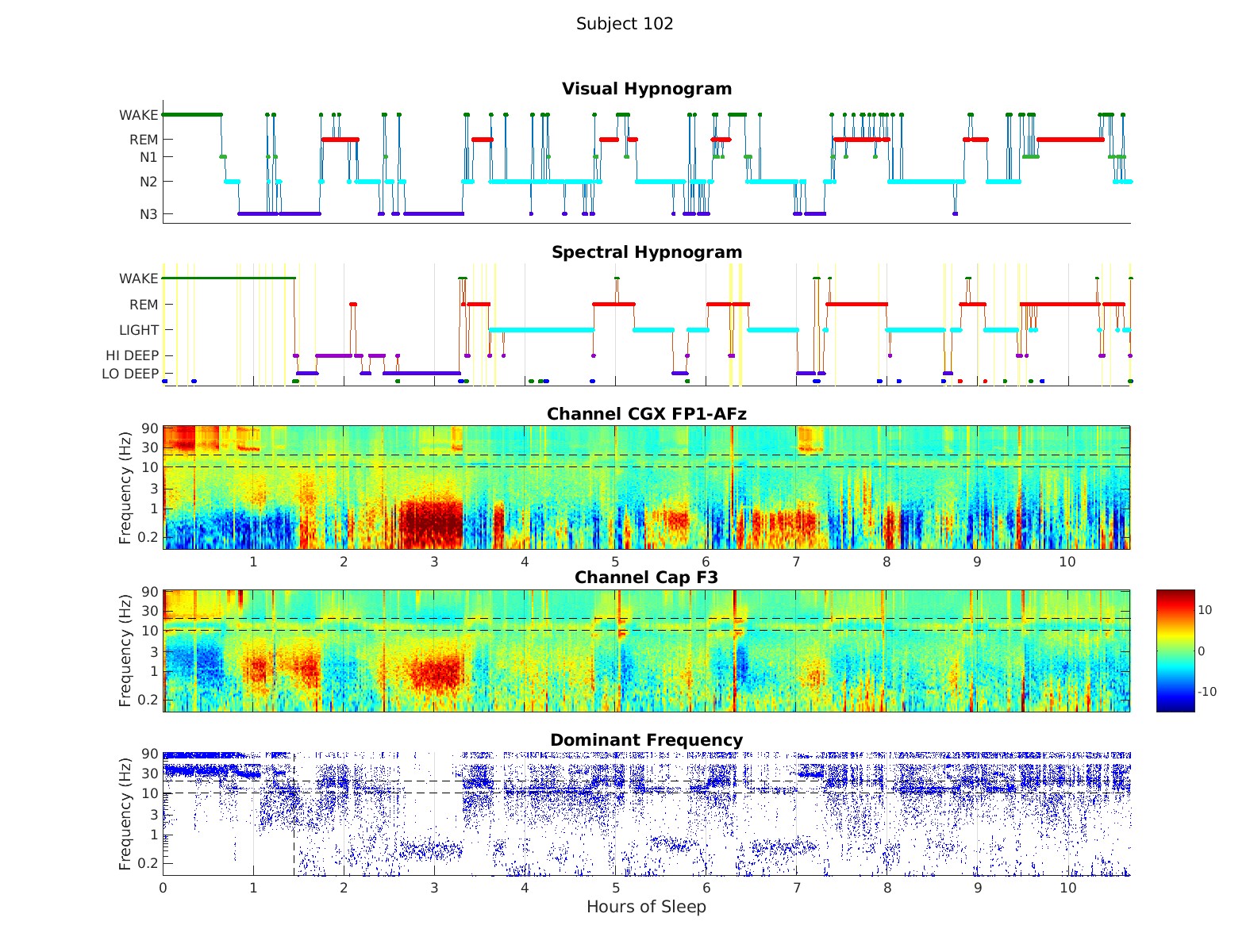

Supplement: Supplementary file 1 [file Data_Sheet_1.ZIP › FP1/102_FP1-F3.jpg]

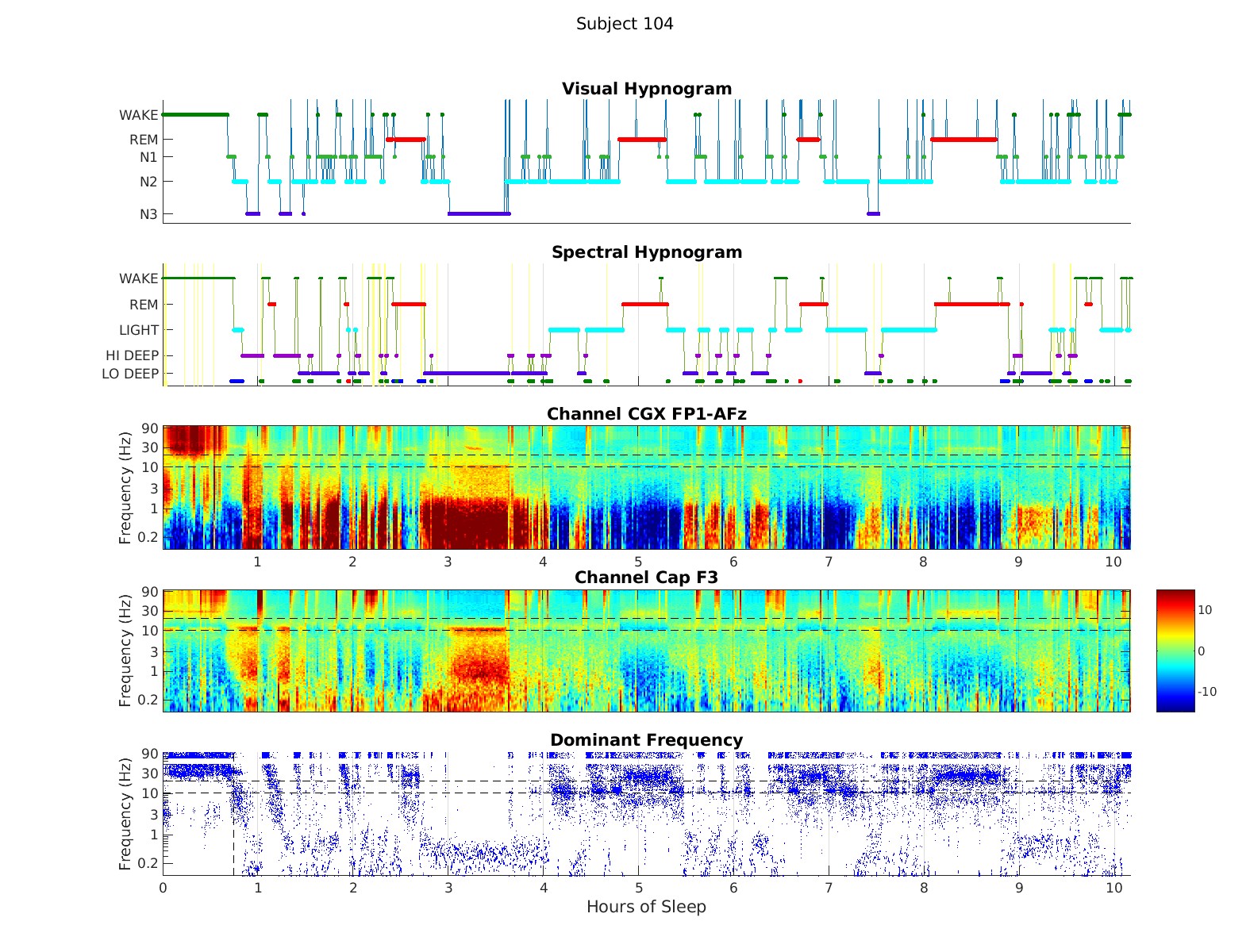

Supplement: Supplementary file 1 [file Data_Sheet_1.ZIP › FP1/104_FP1-F3.jpg]

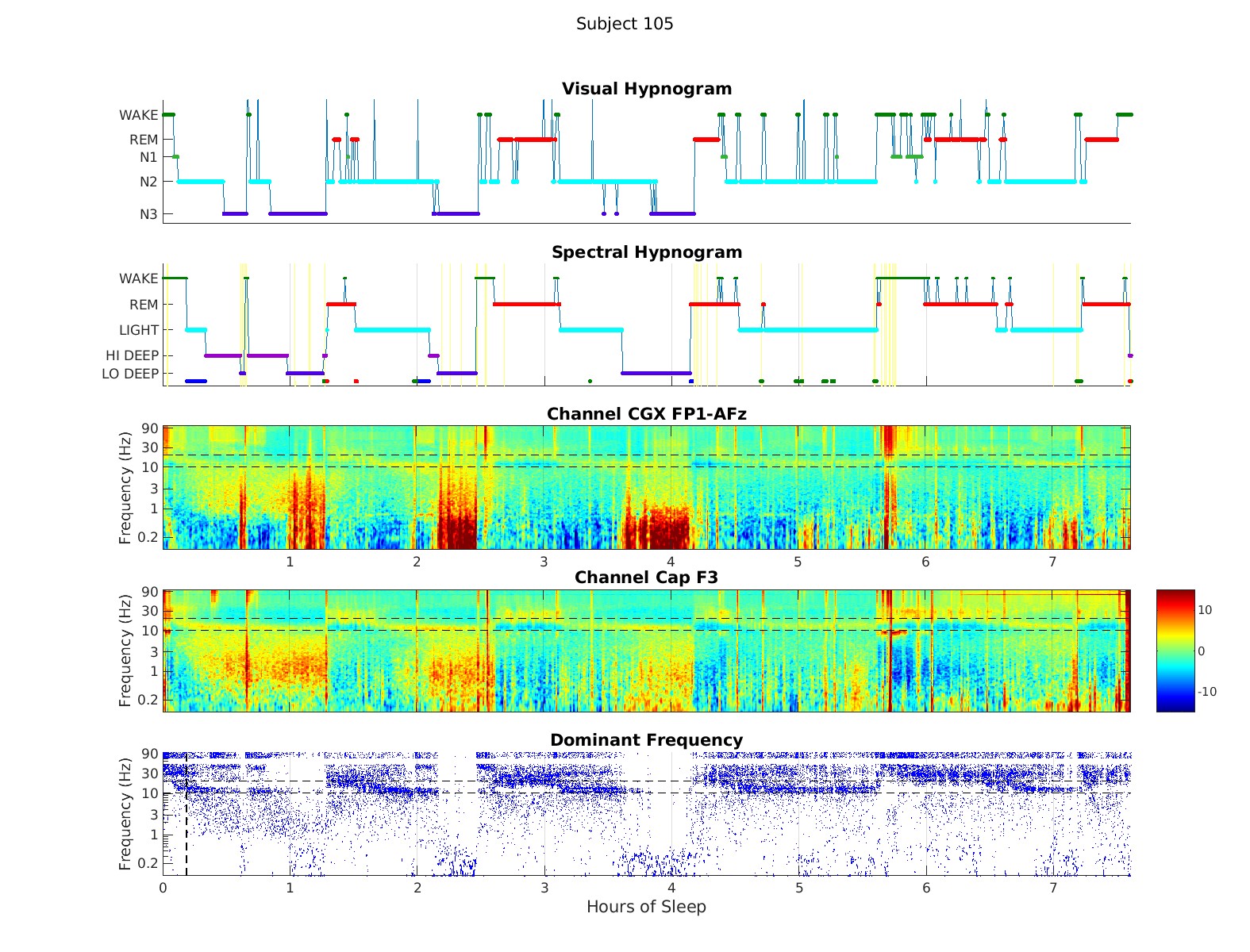

Supplement: Supplementary file 1 [file Data_Sheet_1.ZIP › FP1/105_FP1-F3.jpg]

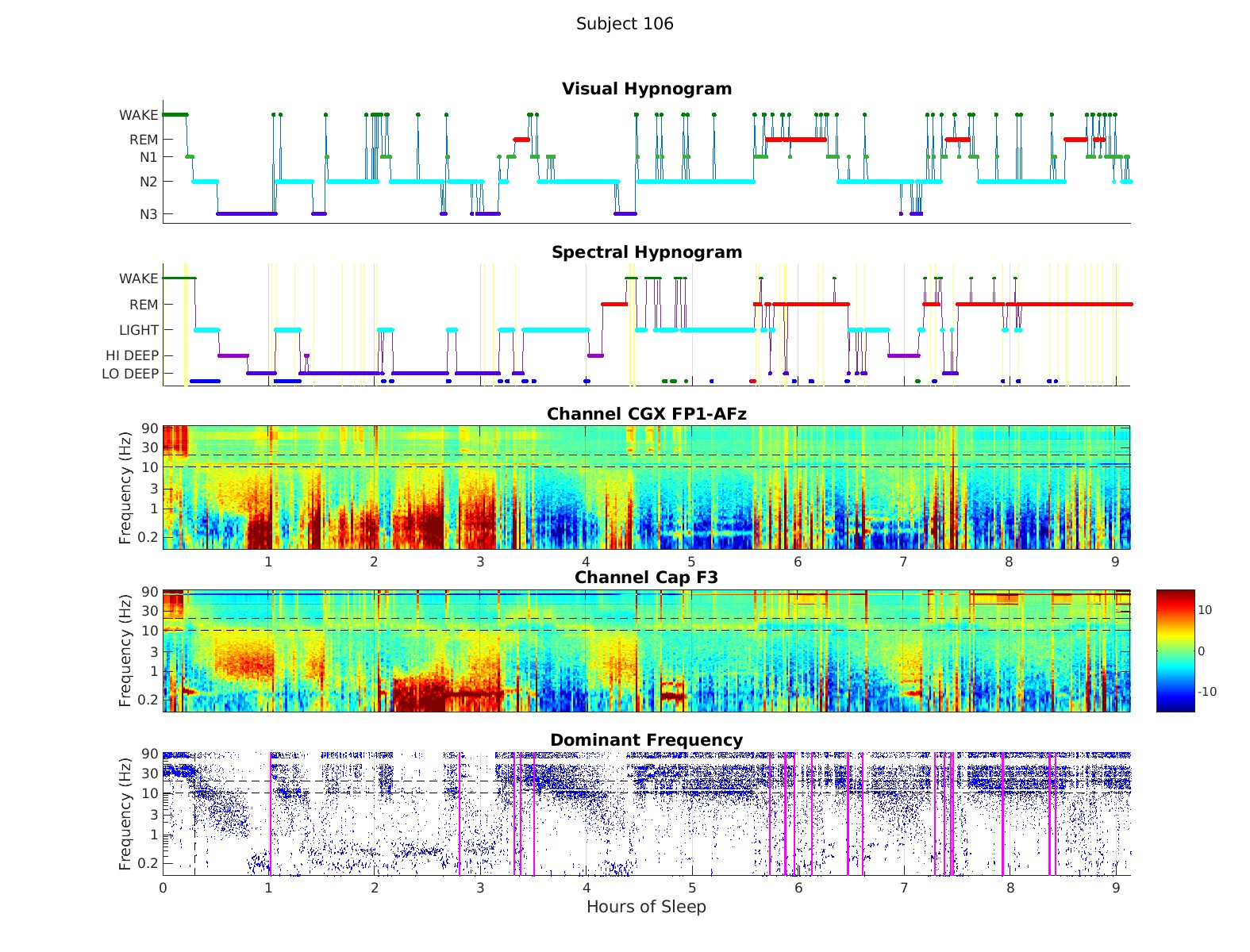

Supplement: Supplementary file 1 [file Data_Sheet_1.ZIP › FP1/106_FP1-F3.jpg]

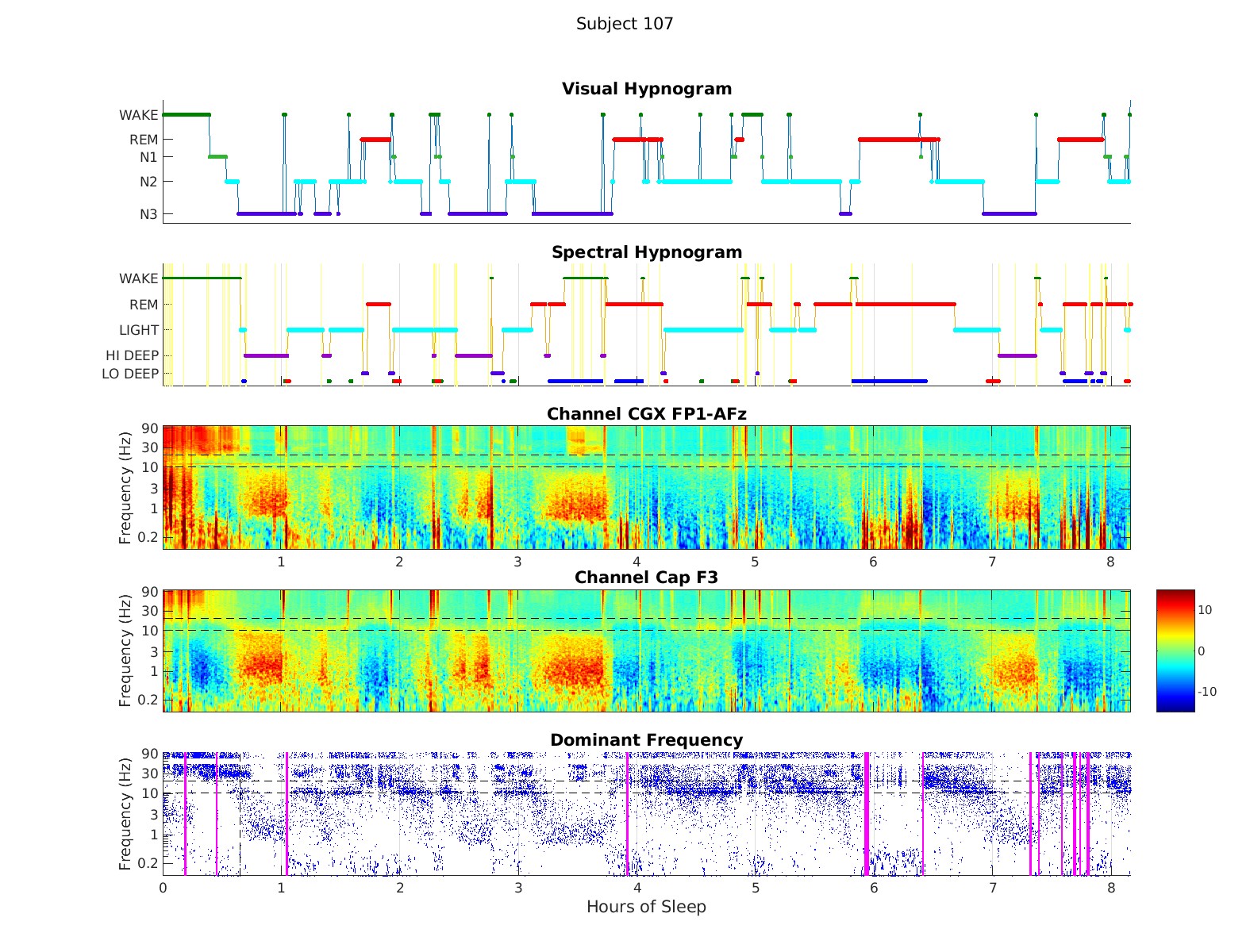

Supplement: Supplementary file 1 [file Data_Sheet_1.ZIP › FP1/107_FP1-F3.jpg]

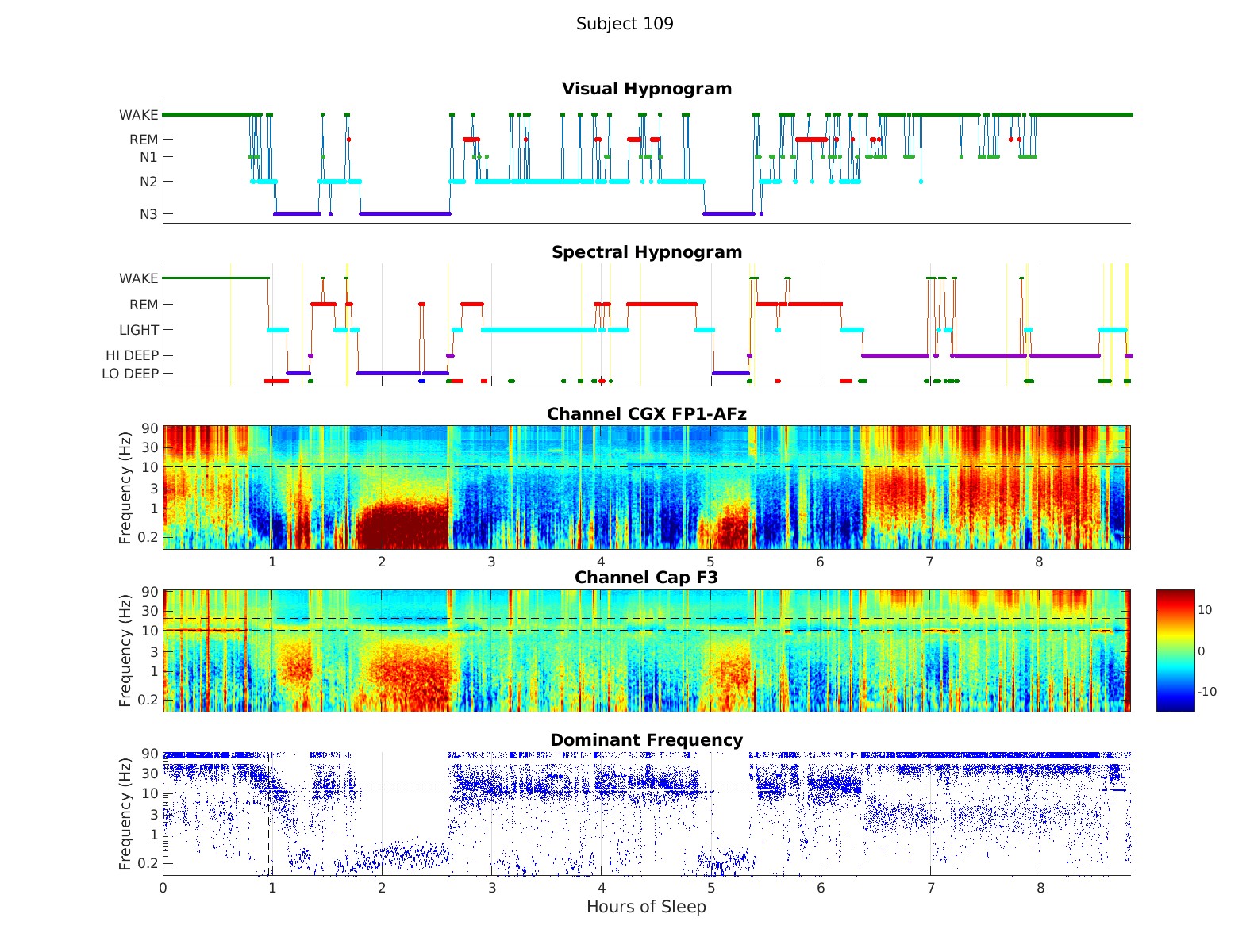

Supplement: Supplementary file 1 [file Data_Sheet_1.ZIP › FP1/109_FP1-F3.jpg]

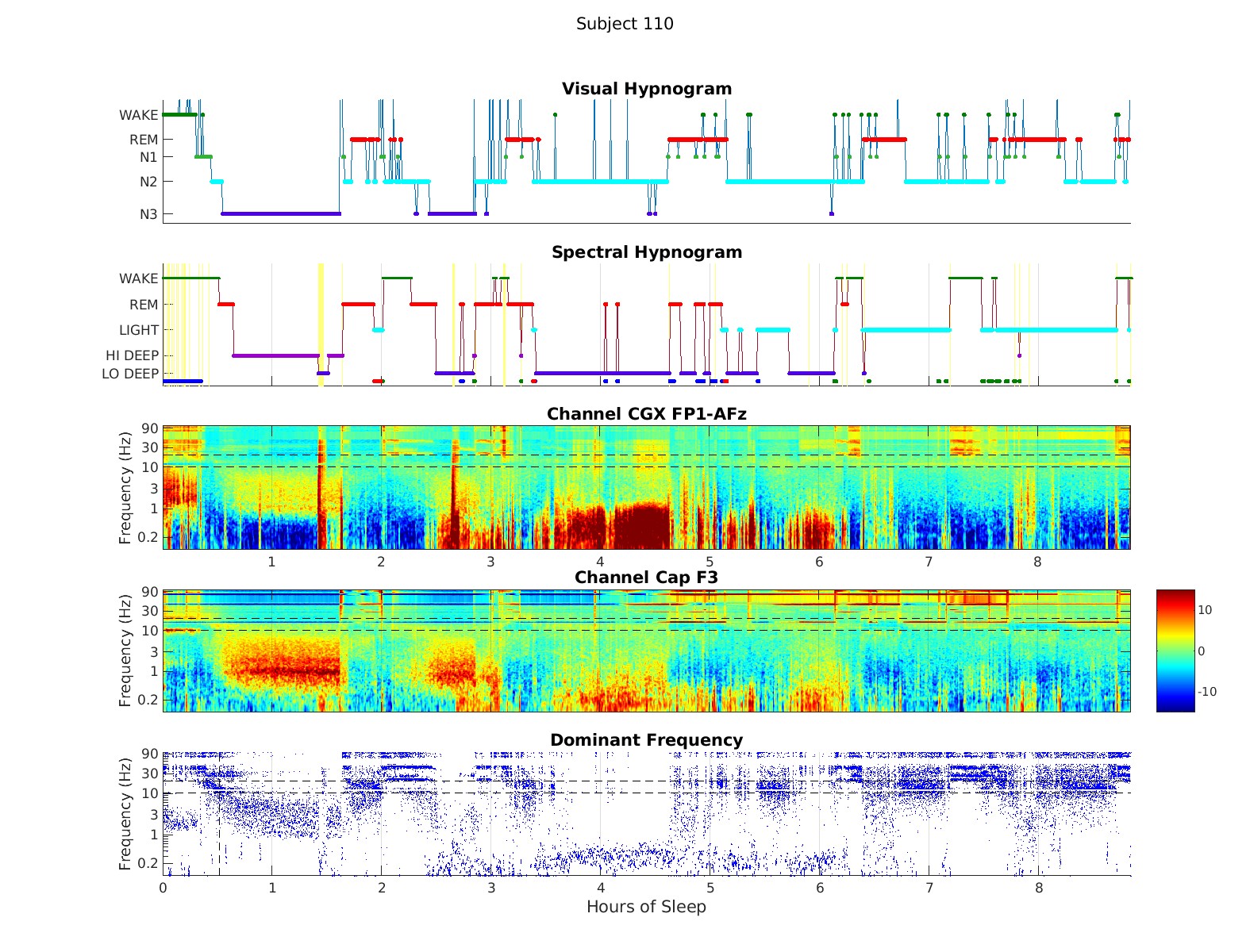

Supplement: Supplementary file 1 [file Data_Sheet_1.ZIP › FP1/110_FP1-F3.jpg]

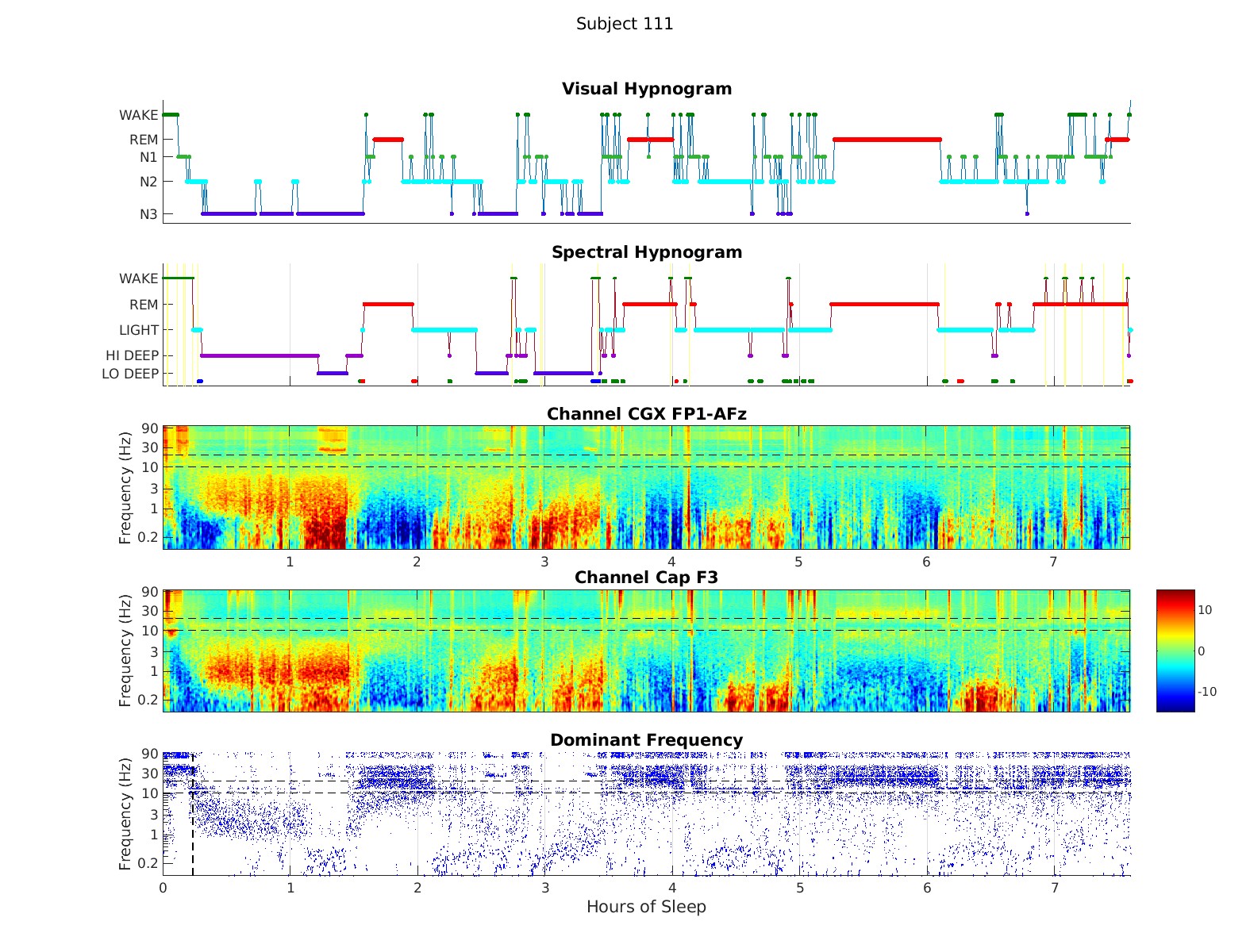

Supplement: Supplementary file 1 [file Data_Sheet_1.ZIP › FP1/111_FP1-F3.jpg]

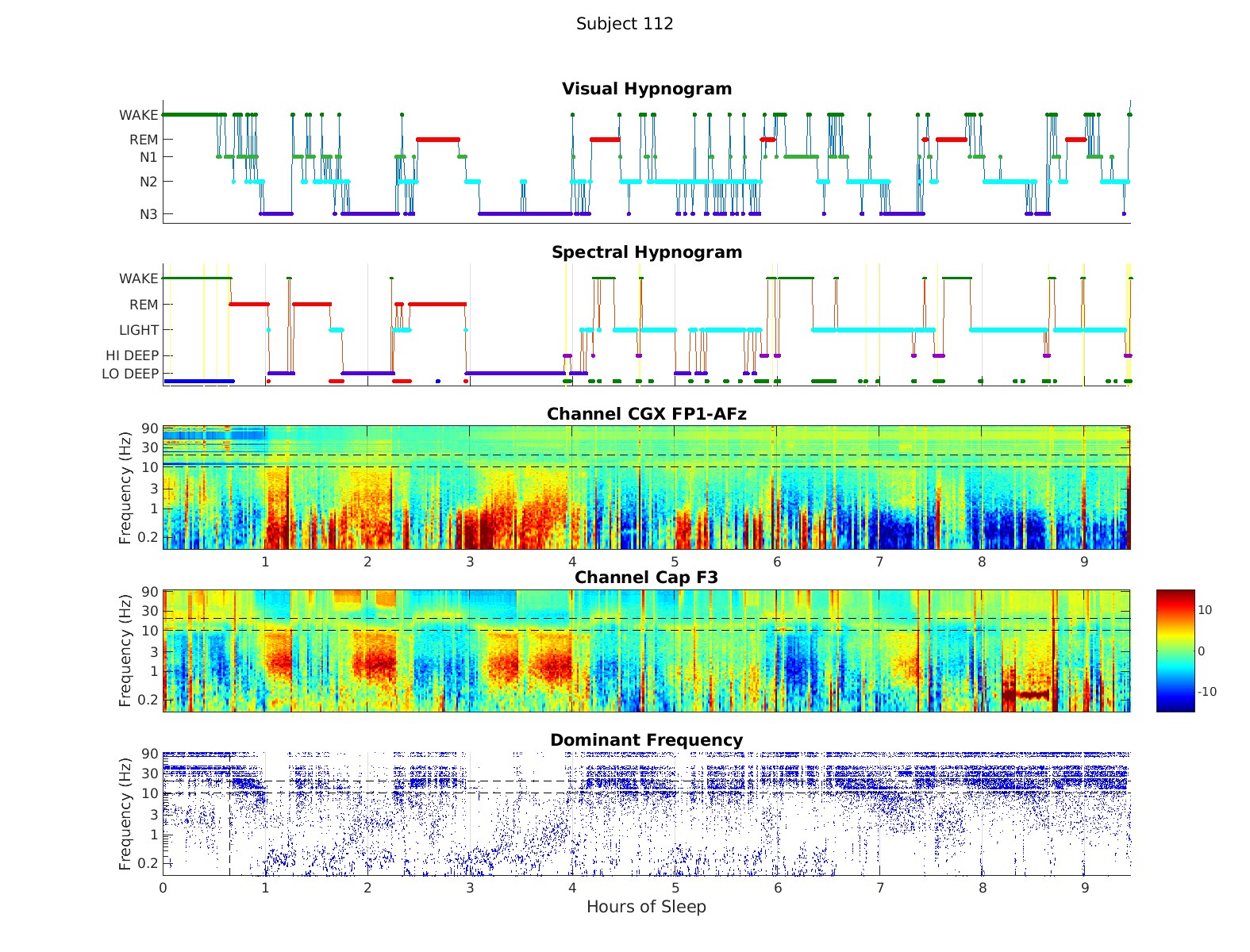

Supplement: Supplementary file 1 [file Data_Sheet_1.ZIP › FP1/112_FP1-F3.jpg]

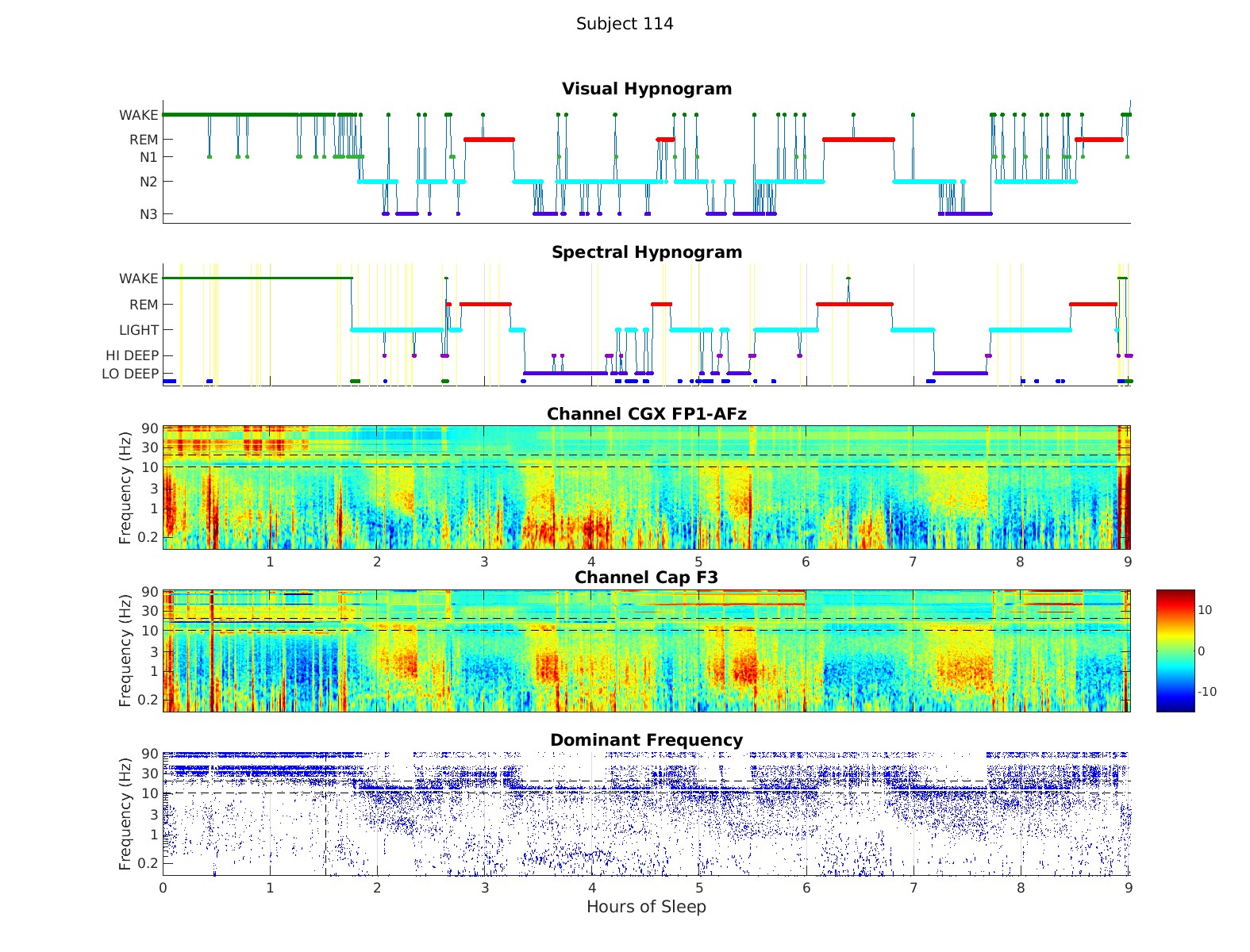

Supplement: Supplementary file 1 [file Data_Sheet_1.ZIP › FP1/114_FP1-F3.jpg]

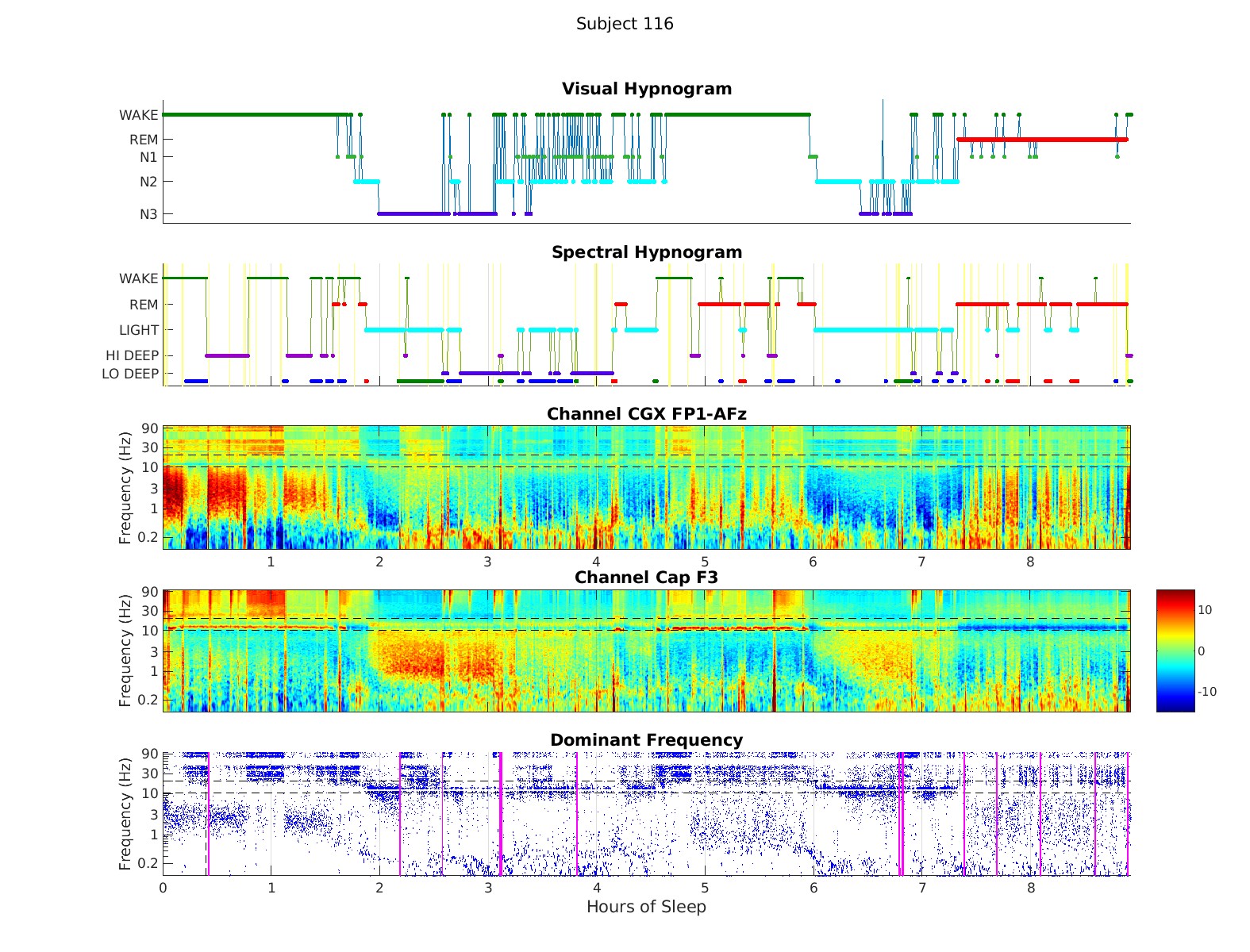

Supplement: Supplementary file 1 [file Data_Sheet_1.ZIP › FP1/116_FP1-F3.jpg]

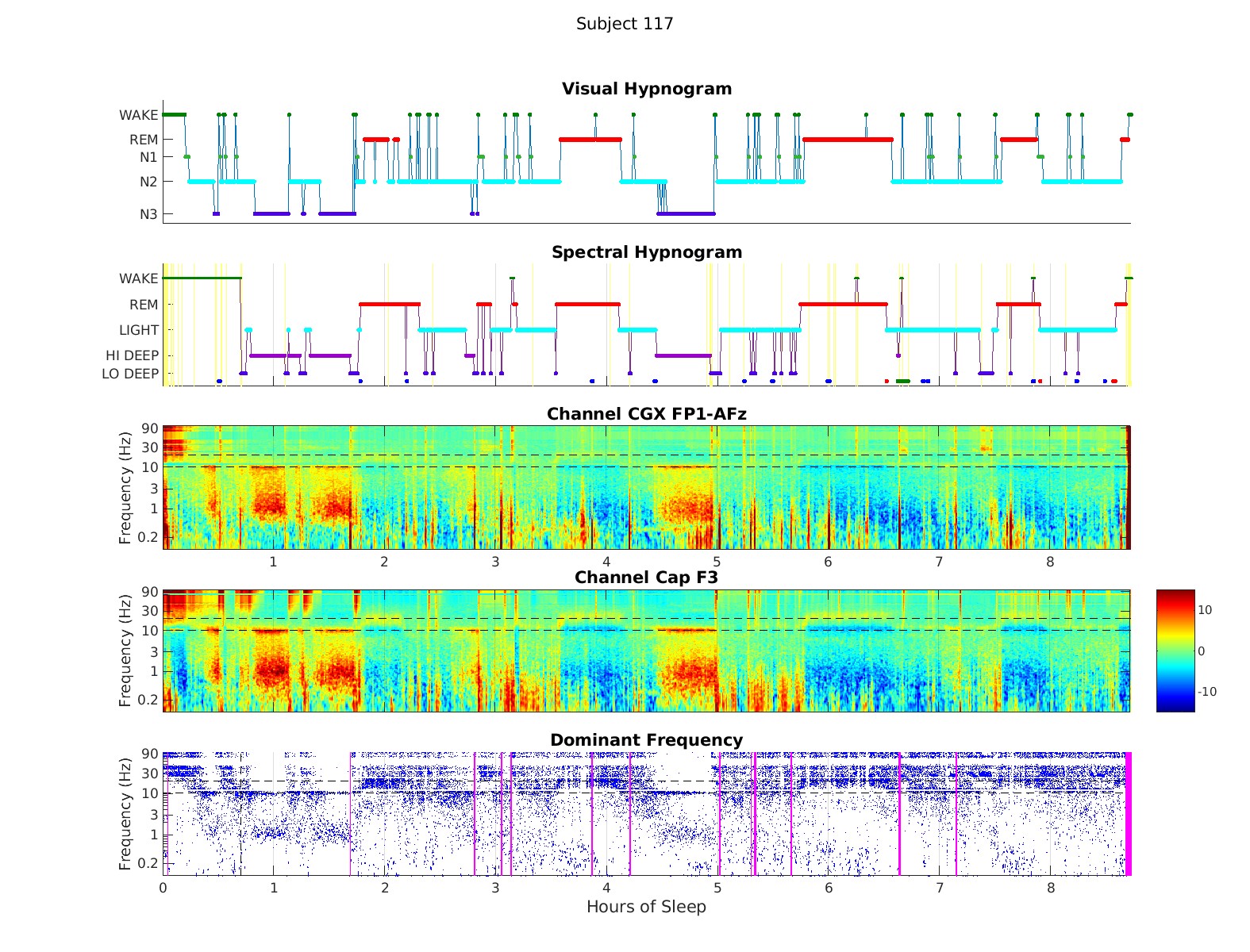

Supplement: Supplementary file 1 [file Data_Sheet_1.ZIP › FP1/117_FP1-F3.jpg]

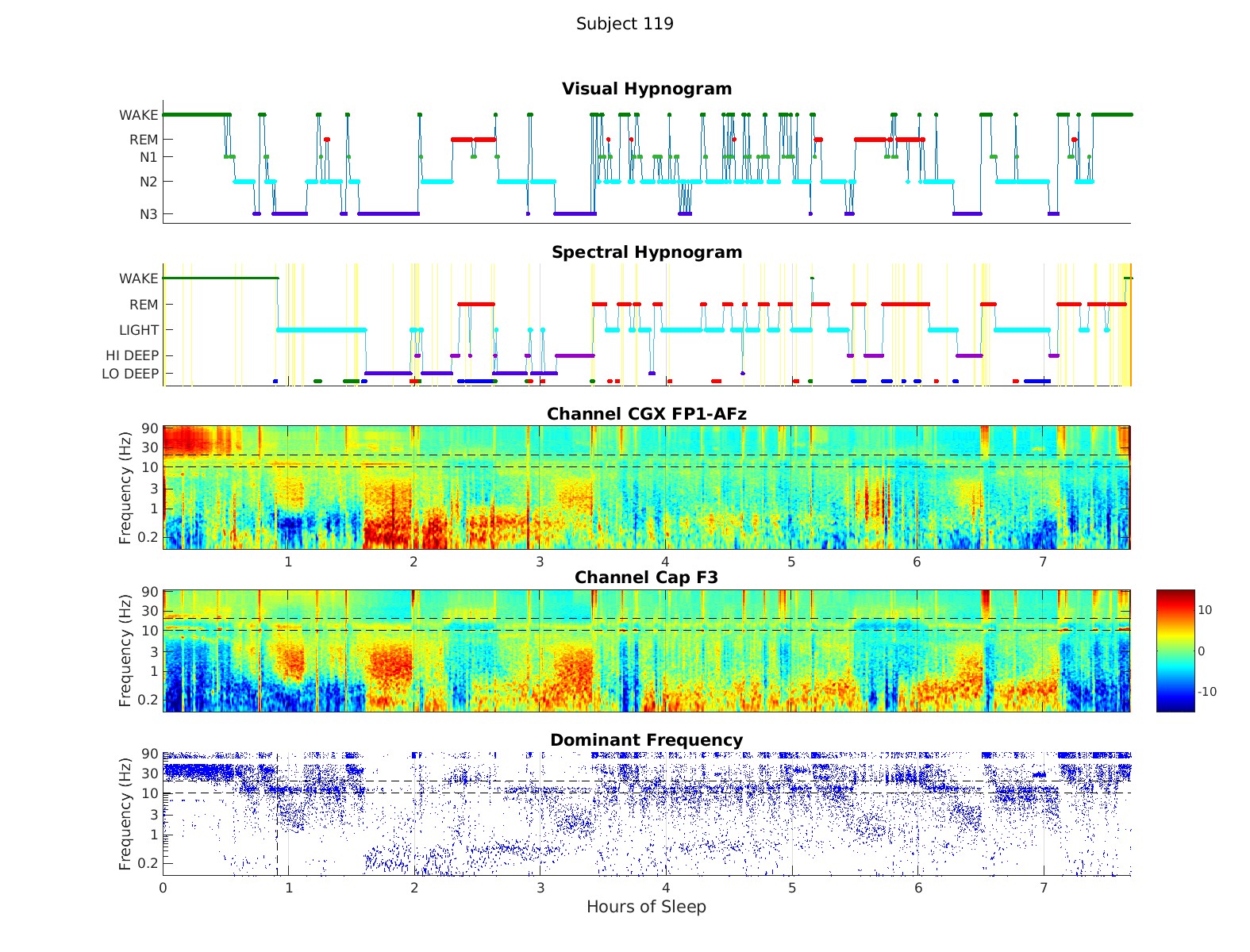

Supplement: Supplementary file 1 [file Data_Sheet_1.ZIP › FP1/119_FP1-F3.jpg]

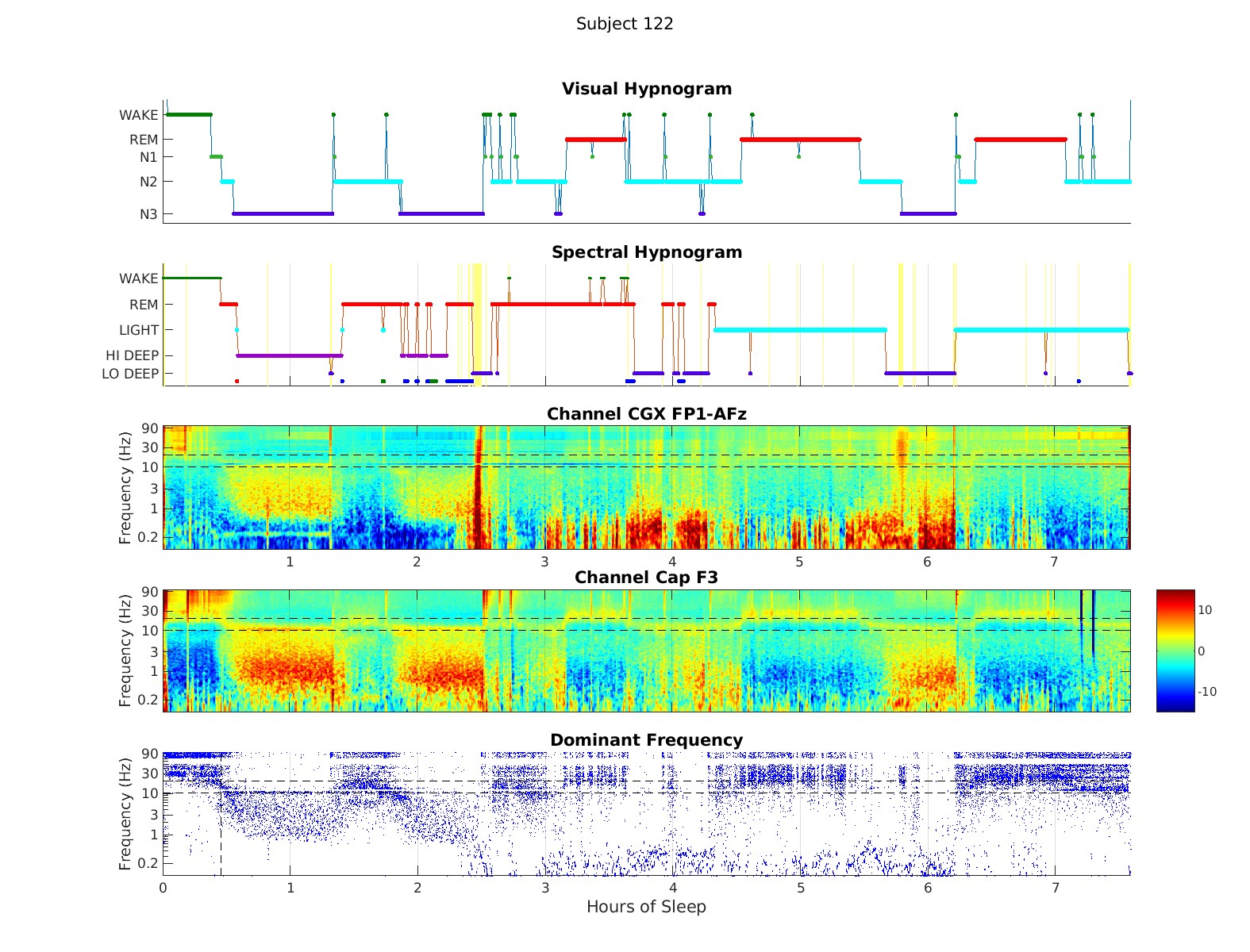

Supplement: Supplementary file 1 [file Data_Sheet_1.ZIP › FP1/122_FP1-F3.jpg]

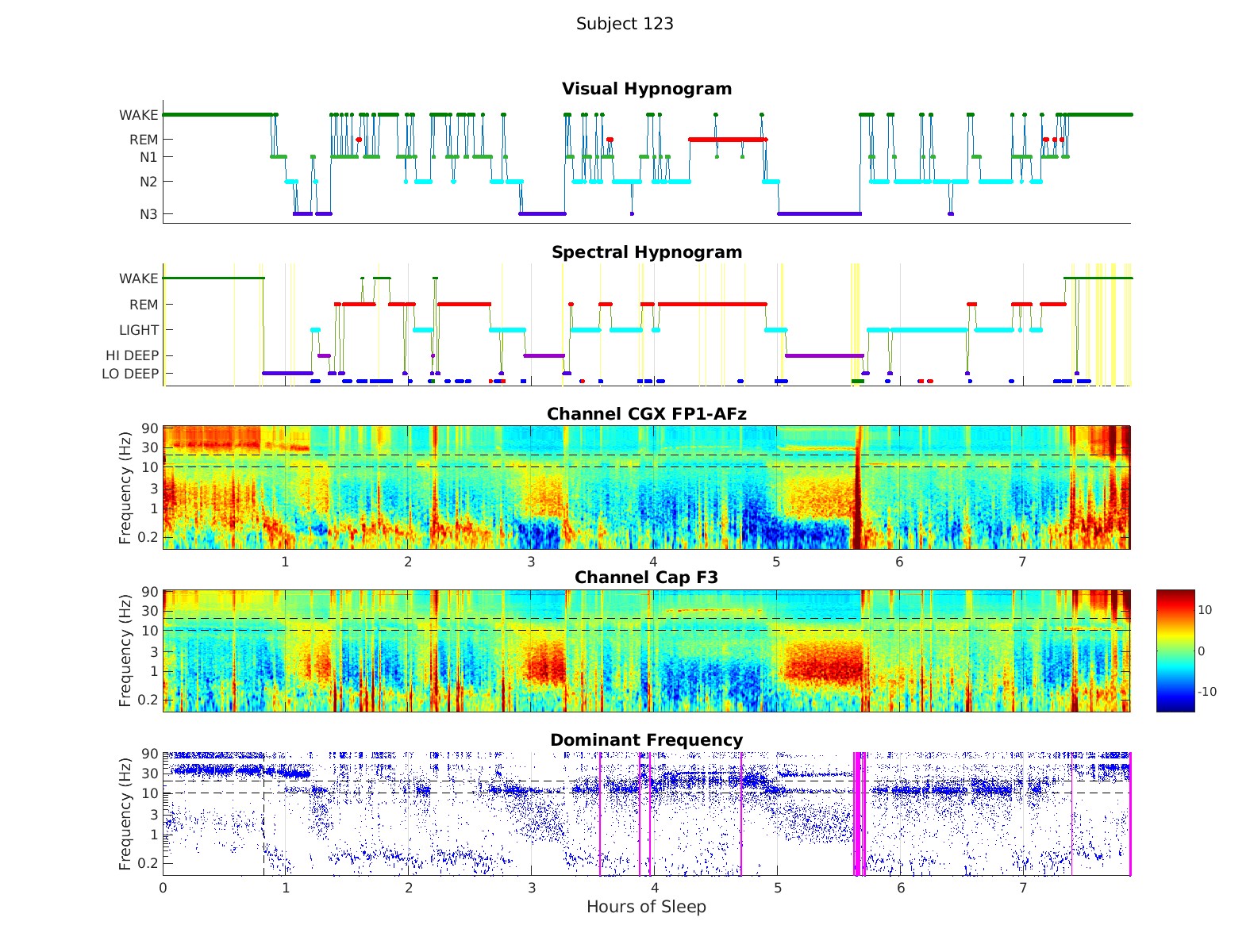

Supplement: Supplementary file 1 [file Data_Sheet_1.ZIP › FP1/123_FP1-F3.jpg]

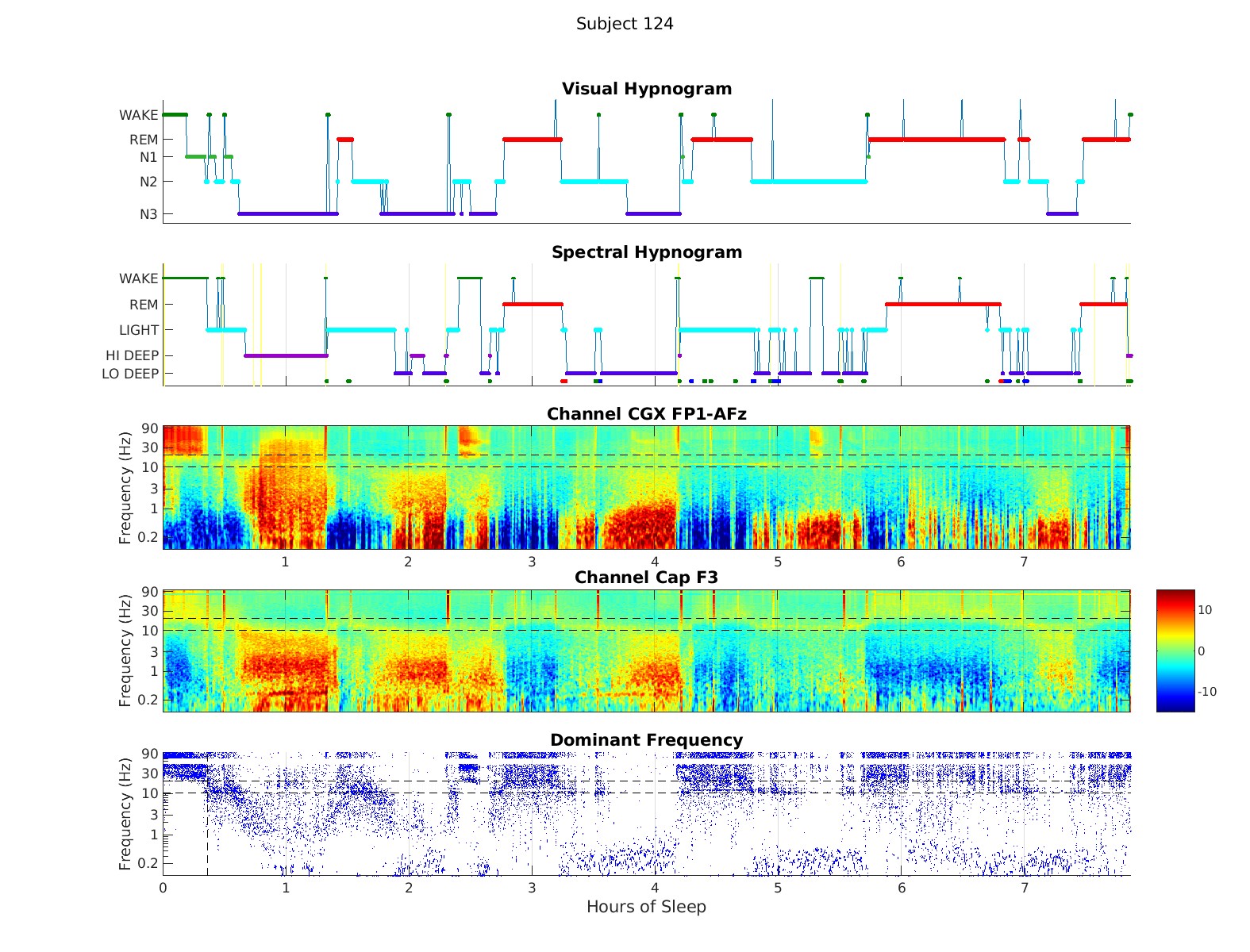

Supplement: Supplementary file 1 [file Data_Sheet_1.ZIP › FP1/124_FP1-F3.jpg]

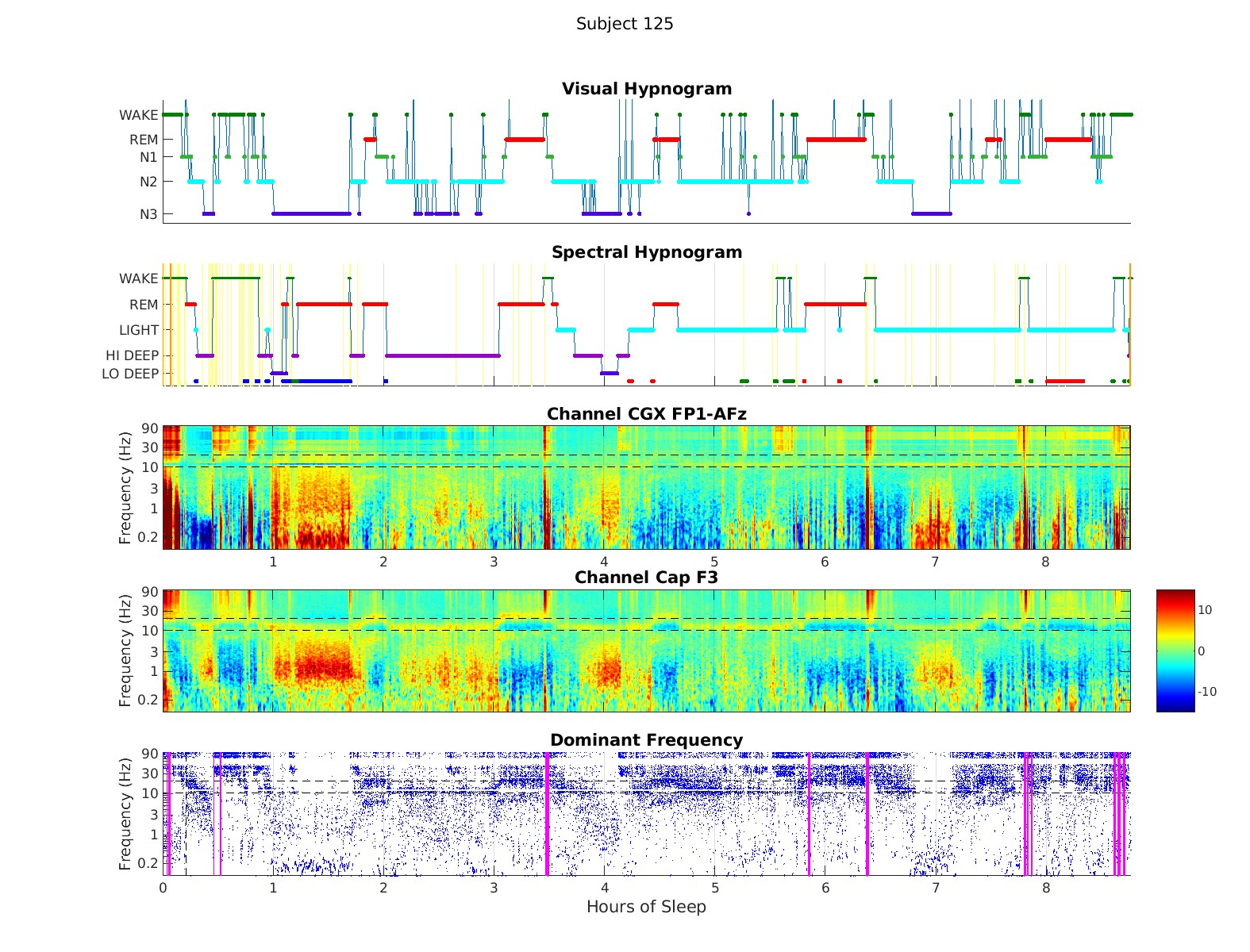

Supplement: Supplementary file 1 [file Data_Sheet_1.ZIP › FP1/125_FP1-F3.jpg]

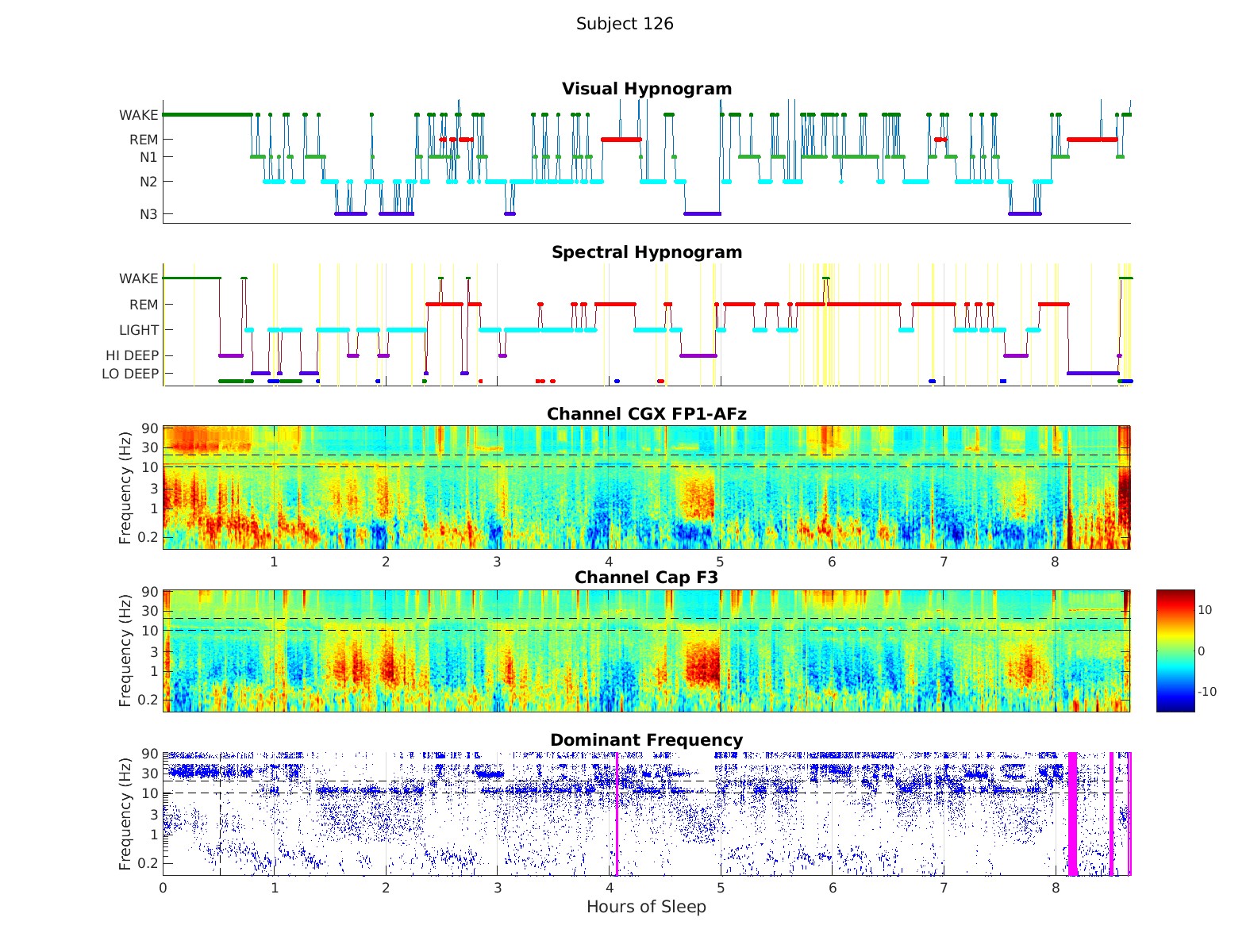

Supplement: Supplementary file 1 [file Data_Sheet_1.ZIP › FP1/126_FP1-F3.jpg]
